# Supplementary material for: Qualitative analysis of stakeholder interviews to identify the barriers and facilitators to the adoption of point-of-care diagnostic tests in the UK
Source: BMJ Open. 2021 Apr 13;11(4):e042944. doi: 10.1136/bmjopen-2020-042944 (PMC8051412; doi:10.1136/bmjopen-2020-042944)
Supplement: Supplementary data [file bmjopen-2020-042944supp001.pdf]

APPENDIX

Appendix 1: POCKET Interview prompt (initial version)

Section 1: Point-of-Care Tests

Clinical Needs Assessment

Example Introductory Statement
"Point-of–care diagnostic tests allow for diagnostic tests to be performed at the bedside, in the clinic or in the patient's home. With advancing diagnostic technologies new point of care tests can give rapid or instant results allowing for real-time clinical decision making in many diseases."

1. What is your opinion of point-of-care diagnostic tests?

   Prompt if required for advantages/disadvantages

2. Where are they required? Why?

3. How can we know where POC diagnostic tests would give most benefit?

   a. (clinical needs assessment/unmet clinical need) Explore unmet needs.

4. Please mark which of the following are useful to determine unmet clinical needs:
   a. Systematic literature reviews
   b. Expert opinion
   c. Patient opinion
   d. Competition analysis
   e. Other

5. Who should be establishing unmet needs?

Section 2: Evidence and Evaluation

1. Once a new point of care diagnostic test has been produced what should the next steps be?

   Prompts: How should we evaluate these devices? Why?

2. What are the goals of evidence generation in new point-of-care diagnostic tests?

1. What evidence best leads to adoption?

2. Do you have any examples of devices that have been adopted easily or with particular difficulty. What caused this? Prompt: home coagulation checking.

3. Please rank the evidence you regard as vital, useful, not required or unsure for the implementation of point of care devices that will be used **in healthcare institutions**?

a. Clinical trials
b. Usability assessment
c. Design Evaluation
d. Patient experience
e. Clinical needs assessment
f. Clinical Pathway Mapping
g. Patient safety/Risk Assessment
h. Economic Analysis
i. Stakeholder Analysis
j. Barriers to adoption
k. Decision Analysis
l. Other

4. Please rank the evidence you regard as vital, useful, not required or unsure for the implementation of point of care devices that will be used **in patient's homes**?

    a. Clinical trials
    b. Usability assessment
    c. Design Evaluation
    d. Patient experience
    e. Clinical needs assessment
    f. Clinical Pathway Mapping
    g. Patient safety/Risk Assessment
    h. Economic Analysis
    i. Stakeholder Analysis
    j. Barriers to adoption
    k. Decision Analysis
    l. Other

5. Where should this evidence come from?
    a. Industry
    b. Academia
    c. Industry/academia partnerships
    d. Consultancy firms
    e. Other

6. What incentives can enhance the adoption of point-of-care test devices?

<u>Validity</u>

Example Introductory Statement
"We will now discuss clinical validity or the accuracy of a diagnostic test."

1. What should evidence on clinical validity demonstrate?

2. Please rank the usefulness of these sources of evidence for clinical validity?

    a. Randomised controlled trial evidence
    b. Diagnostic Accuracy Studies (Case-control)
    c. Linked Evidence Approaches
    d. Bayesian Networks
    e. Other approach

3. What if there is no reference standard?

   "Point-of-care tests are often seen as a disruptive innovation, by which I mean that the introduction of the test disturbs the established clinical pathway. This can be demonstrated by clinical pathway modeling to predict the impact of implementation."

4. Should new tests be modeled against current practice or best practice?

5. How translatable is this evidence between different institutions or different healthcare systems?

6. Is a trade off in accuracy acceptable for the convenience of point-of-care tests?

7. Any other thoughts regarding the accuracy or validity of POC tests?

<u>Usability</u>

Introductory Statement
"Point-of-care tests are often performed away from central laboratories or in the patient's home and therefore trained laboratory personnel are not present and those using the test will have limited training. Therefore, these devices need to be user-friendly."

1. What are your thoughts on the usability of point-of care diagnostic test?

2. Should this be tested? How?

3. Is evidence demonstrating the usability of a device important for implementation?

4. How should this be presented?

5. What usability assessments are you aware of?

6. Is evidence on usability required that is over and above what is required for regulatory approval (FDA/CE marking)?

7. How does patient safety impact on implementation of new tests?

   a. Prompt if required re risk assessment

8. Any other thoughts on the usability assessment of point-of-care diagnostic tests?

<u>Economic Analysis</u>

Introductory Statement
"Healthcare budgets are a finite resource and cost-effectiveness needs to be demonstrated to justify the use of new diagnostic tests."

1. What do you think gives value to new point-of-care diagnostic tests?

2. How is this measured?

3. What economic information would benefit the implementation of point-of-care diagnostic tests?

4. Are modeling techniques reliable? Why?

7. Are quality adjusted life years (QALY) a useful and sufficient measurement of value in the evaluation of diagnostic tests?

8. Any other thoughts regarding the economic analysis of point-of-care diagnostic tests?

<u>Patient Experience</u>

Introductory statement
"Patients are the reason why diagnostic tests are performed so when engaging with patients regarding new technologies…"

1. How should patient experience and opinion be incorporated into the evaluation of new point-of-care diagnostic tests?

2. What evidence should patients have in new diagnostic tests?

<u>Section 3: Adoption</u>

<u>Barriers</u>

Example Introductory statement
"Even with good evidence some point-of care-technologies fail or are slow to be implemented."

1. What are the challenges in generating evidence to justify the implementation of new point-of-care diagnostic tests?

1. How can these be overcome?

2. Is there any other evidence you feel would assist in the implementation of new point-of-care diagnostic tests?

3. Are there any other barriers to the implementation of new point-of-care diagnostic tests?

4. What can the NIHR Diagnostic Evidence Co-operatives do to help you make decisions?

5. NICE/Industry/CCG: As a partner to the Diagnostic Evidence Co-operatives, how do you envisage us working together?

<u>Efficient Evidence Generation</u>

Example Introductory Statement
“Effective study design may allow evidence in differing domains to be gathered more efficiently.”

1. How can we be more efficient in the evaluation process of point-of-care diagnostic tests?

2. What changes would you like to see?

3. Are you aware of Bayesian Networks being used to combine sources of evidence?

4. Any other thoughts or comments regarding point-of-care tests and evidence generation?
