# Supplementary material for: Qualitative analysis of stakeholder interviews to identify the barriers and facilitators to the adoption of point-of-care diagnostic tests in the UK
Source: BMJ Open. 2021 Apr 13;11(4):e042944. doi: 10.1136/bmjopen-2020-042944 (PMC8051412; doi:10.1136/bmjopen-2020-042944)
Supplement: Author's manuscript [file bmjopen-2020-042944.draft_revisions.pdf]

BMJ Open is committed to open peer review. As part of this commitment we make the peer review history of every article we publish publicly available.

When an article is published we post the peer reviewers' comments and the authors' responses online. We also post the versions of the paper that were used during peer review. These are the versions that the peer review comments apply to.

The versions of the paper that follow are the versions that were submitted during the peer review process. They are not the versions of record or the final published versions. They should not be cited or distributed as the published version of this manuscript.

BMJ Open is an open access journal and the full, final, typeset and author-corrected version of record of the manuscript is available on our site with no access controls, subscription charges or pay-per-view fees (http://bmjopen.bmj.com).

If you have any questions on BMJ Open's open peer review process please email info.bmjopen@bmj.com

BMJ Open

Barriers and Facilitators to the Adoption of Point-of-Care Diagnostic Tests

Journal:	*BMJ Open*
Manuscript ID	bmjopen-2020-042944
Article Type:	Original research
Date Submitted by the Author:	23-Jul-2020
Complete List of Authors:	Huddy, Jeremy; Imperial College London, Surgery and Cancer<br>Ni, Melody; Imperial College London, Surgery and cancer<br>Barlow, James; Imperial College London, Business School<br>Hanna, George; Imperial College London, Faculty of Medicine, Department of Surgery & Cancer
Keywords:	CHEMICAL PATHOLOGY, Health economics < HEALTH SERVICES ADMINISTRATION & MANAGEMENT, Health policy < HEALTH SERVICES ADMINISTRATION & MANAGEMENT, Clinical chemistry < PATHOLOGY

SCHOLARONE™
Manuscripts

I, the Submitting Author has the right to grant and does grant on behalf of all authors of the Work (as defined in the below author licence), an exclusive licence and/or a non-exclusive licence for contributions from authors who are: i) UK Crown employees; ii) where BMJ has agreed a CC-BY licence shall apply, and/or iii) in accordance with the terms applicable for US Federal Government officers or employees acting as part of their official duties; on a worldwide, perpetual, irrevocable, royalty-free basis to BMJ Publishing Group Ltd ("BMJ") its licensees and where the relevant Journal is co-owned by BMJ to the co-owners of the Journal, to publish the Work in this journal and any other BMJ products and to exploit all rights, as set out in our licence.

The Submitting Author accepts and understands that any supply made under these terms is made by BMJ to the Submitting Author unless you are acting as an employee on behalf of your employer or a postgraduate student of an affiliated institution which is paying any applicable article publishing charge ("APC") for Open Access articles. Where the Submitting Author wishes to make the Work available on an Open Access basis (and intends to pay the relevant APC), the terms of reuse of such Open Access shall be governed by a Creative Commons licence – details of these licences and which Creative Commons licence will apply to this Work are set out in our licence referred to above.

Other than as permitted in any relevant BMJ Author's Self Archiving Policies, I confirm this Work has not been accepted for publication elsewhere, is not being considered for publication elsewhere and does not duplicate material already published. I confirm all authors consent to publication of this Work and authorise the granting of this licence.

For peer review only

Barriers and Facilitators to the Adoption of Point-of-Care Diagnostic Tests

Jeremy R Huddy (PhD FRCS),[1] Melody Ni (PhD),[1] James Barlow (PhD),[2] George B Hanna (PhD, FRCS)[1]

[1]Department of Surgery and Cancer, Imperial College, London, UK

[2]Imperial College Business School, South Kensington, London, UK

The National Institute for Health Research Diagnostic Evidence Co-operative London at Imperial College Healthcare NHS Trust

St Mary's Hospital, South Wharf Road, London, W2 1NY, United Kingdom

Correspondence to:
Professor George B Hanna
Department of Surgery and Cancer
Imperial College London
Academic Surgical Unit, 10th Floor, QEQM Building
St Mary's Hospital, South Wharf Road
London, W2 1NY, United Kingdom
Tel: +44 203 312 2124
Fax: +44 203 312 6309
Email: g.hanna@imperial.ac.uk

Article category: Research Article

Word Count: 4465 words

Abstract: 215 words

Tables: 2

Figures: 8 text boxes

For peer review only

For peer review only - http://bmjopen.bmj.com/site/about/guidelines.xhtml

Funding: This research was supported by the National Institute for Health Research (NIHR) Diagnostic Evidence Co-operative London at Imperial College Healthcare NHS Trust. The views expressed are those of the authors and not necessarily those of the NHS, the NIHR or the Department of Health.

Keywords: Point-of-Care Testing

Patient and Public Involvement: The National Institute for Health Research (NIHR) Diagnostic Evidence Co-operative London at Imperial College Healthcare NHS Trust has an active patient and public involvement group who inform the identification, prioritisation, design, and execution of our research (https://london.ivd.nihr.ac.uk/patient-public/).

Competing Interests: The authors declare no competing interest.

Data availability: No data are available.

.

For peer review only - http://bmjopen.bmj.com/site/about/guidelines.xhtml

ABSTRACT

Objectives: This study investigated the barriers and facilitators to the adoption of point-of-care tests (POCTs).

Design: Qualitative study incorporating a constant comparative analysis of stakeholder responses to a series of interviews undertaken to design the Point-of-Care Key Evidence Tool (POCKET).

Setting: The study was conducted in relation to POCTs used in all aspects of healthcare.

Participants: Forty-three stakeholders were interviewed in five groups that included clinicians (incorporating laboratory staff and members of trust POCT committees), commissioners, industry, regulators and patients.

Results: Thematic analysis highlighted 32 barriers in six themes and 28 facilitators in eight themes to the adoption of POCTs. Six themes were common to both barriers and facilitators (clinical, cultural, evidence, design and quality assurance, financial and organisational) and two themes contained facilitators alone (patient factors and other (non-financial) resource use).

Conclusions: Findings from this study demonstrate the complex motivations of stakeholders in the adoption of POCT. Most themes were common to both barriers and facilitators suggesting that good device design, stakeholder engagement and appropriate evidence provision can increase the likelihood of a POCT device adoption. However, it is important to realise that whilst the majority of identified barriers may be perceived or mitigated some may be absolute and if identified early in device development the value of further investment should be carefully considered.

For peer review only

STRENGTHS AND LIMITATIONS

- Semi-structured interview study incorporated a diverse range of views from stakeholders involved in the implementation of POCT
- Thematic analysis identified the barriers and facilitators that should be considered when developing POCT
- Interview topic guide grouped all POCT together and incentives and disincentives may vary between different POCT categories (eg. patient self-testing, testing in primary care etc.)

INTRODUCTION

Point-of-care tests (POCT) are tests that are carried out in close proximity to patients by non-laboratory trained personnel either in healthcare settings or the home. Technological advances have led to more tests traditionally performed in clinical laboratories being available to clinicians at the bedside.[1] This convenience can allow for real time decision-making within patient care pathways and has led to improved access to diagnostics, reduced time to treatments, patient satisfaction and cost savings.[2]

Healthcare systems represent significant challenges to technology adoption. It is often not failures in the technology but cultural, organisational and financial aspects of the system that prevent implementation. The diffusion of innovation traditionally follows an S-shaped curve as an innovation passes through stages of adoption and spread before plateauing at mainstream adoption.[3] However, the length of time taken innovations to be adopted in health systems has been well documented around the world; one review suggests an average of 17 years.[4] Efforts need to be made to speed up this process and identify factors that can hamper or accelerate the adoption journey so that the benefits to patients and society of these devices can be realised.

For peer review only - http://bmjopen.bmj.com/site/about/guidelines.xhtml

Barriers and facilitators can be reliably studied using qualitative techniques.[5–7] A better understanding of the adoption motivations for POCTs may inform strategies to mitigate barriers and promote facilitators that expediate the time it takes for devices to become integrated into patient pathways. Barriers and facilitators to healthcare technologies were investigated as part of the 2016 Accelerated Access Review (AAR) commissioned by the UK government and supported by the Wellcome Trust).[8] This review began with an interim report that highlighted a list of barriers that stand in the way of innovation adoption in healthcare systems. However, the findings from this report reflect a wide variety of healthcare technology including diagnostics, medical devices and pharmaceuticals findings and therefore may not represent the specific challenges of scaling up POCT technology. Given the increasing number of POCTs available and their potential impact across clinical pathways there is value in investigating the specific barriers and facilitators to POCT technology adoption. This would allow better understanding of the potential challenges in commercializing and implementing POCT tests, so that modifications may be made early in their development and guide the collection of evidence needed to meet address the needs of clinical and other stakeholders. Two systematic reviews have previously investigated such barriers; in their systematic narrative review of the barriers to hospital-based adoption of POCT *Quinn et al* identified the most prevalent barrier categories being the economics of adoption, quality assurance and regulatory issues, device performance and data management and staff and operation issues.[9] *Jones et al* investigated the attitudes of primary care clinicians in Europe and Australia towards POCTs and reported the three main barriers being related to the impact on clinical practice, impact on patient-clinician relationships and perceived patient experience.[10]

Previous work has led to the development of the Point-of-Care Key Evidence Tool (POCKET) tool [11] that reports the evidence that is a required by different stakeholder groups to facilitate good decision making in regards the adoption of

For peer review only - http://bmjopen.bmj.com/site/about/guidelines.xhtml

POCT. As part of this study a series of in-depth semi-structured interviews was undertaken with a broad range of relevant stakeholders. The aim of this study was to undertake a qualitative analysis of these interviews with a specific focus on identifying the barriers and facilitators that exist to the adoption of POCTs within patient pathways.

METHODS

A qualitative analysis of the 41 semi-structured stakeholder interviews that were undertaken to develop POCKET was undertaken using grounded theory. The study description and results are summarised in accordance with the Consolidated Criteria for Reporting Qualitative Research (COREQ) checklist.[12] Included stakeholders were clinicians (including laboratory staff and members of trust POCT committees), commissioners, industry, regulators and patients. The methodology for the development of the POCKET checklist including the interview phase, topic guide and recruitment strategy has been previously published.[11,13] The interviews were undertaken by one author (JRH). The interview participants received information relating to the study in advance of the interview through a participant information leaflet. The interviews were semi-structured and a topic list was prepared in advance and piloted (appendix 1). The interview topic guide included a section relating to adoption where barriers and facilitators were explored. Interviews were undertaken either in person, by telephone or through Skype (Microsoft, Seattle, WA, USA). Interviews were recorded. Following verbatim transcription transcripts were not returned to participants for verification. Interviews were analysed by two researchers (JRH and a clinical research nurse working within the NIHR Diagnostic Evidence Co-operative London) who independently reviewed the transcripts using a constant comparative technique before meeting to compare emergent themes. Interviews were undertaken until saturation had been reached, as demonstrated by the absence of new themes emerging from the analysis. NVivo (version 11.2.2 QSR International)

For peer review only - http://bmjopen.bmj.com/site/about/guidelines.xhtml

was used to query the data and provide matrix coding so that the frequency of stakeholders in each group who identified a particular barrier or facilitator could be determined.

RESULTS

A total of 41 semi-structured interviews were undertaken with 43 stakeholders (two interviews were undertaken with two participants concurrently at the request of the participants) between August 2014 and December 2015. This included 5 stakeholder groups (regulators [n = 10], industry [n = 9], commissioners [n = 8], clinicians [including doctors, laboratory staff and point-of-care committee members] [n = 8], patients [n = 8]). Recruitment rates from invitation were 80% for clinicians and 29% for commissioners. Regulators, industry representatives and patients were recruited through bodies external to the study group and recruitment rate is not known. Twenty-three (55%) interviews were undertaken face to face and 19 (45%) by Skype (Microsoft, Seattle, WA, USA) or telephone. Interviews had a median length of 31 minutes (range 12-58 minutes). For the purpose of reporting interviewees are referred to as stakeholders.

Thematic analysis highlighted 32 barriers in six themes and 28 facilitators in eight themes to the adoption of POCTs. Six themes were common to both barriers and facilitators (clinical, cultural, evidence, design and quality assurance, financial and organisational) and two themes contained facilitators alone (patient factors and other (non-financial) resource use). Tables 1 and 2 summarise the barriers and facilitators identified within each theme and the number of participants from each stakeholder group who identified each. The sections below qualitatively summarise the themes that emerged during the interviews along with verbatim quotations.

Clinical Barriers and Facilitators

For peer review only - http://bmjopen.bmj.com/site/about/guidelines.xhtml

The benefits that POCTs are able to bring to clinical pathways were widely recognised by all stakeholders. Particular focus was given to the communication benefits, better patient care, improved turnaround time and effective patient triage. However, some POCTs were felt to be available without a defined clinical need or pathway benefit and these were thought to be less likely to succeed. Furthermore, even when a clinical need was determined, given the volume and accuracy offered if a laboratory was able to deliver a test result at a time point that doesn't affect the efficacy of the associated intervention then the POCT's utility can still be questioned (unless other benefits can be demonstrated). Some stakeholders were concerned by the inappropriate use of POCTs, for example patients self-testing too often or when not indicated by clinicians. POCTs were seen by some to discourage the use of clinical judgement, leading to an overreliance on guidelines and treatment algorithms; conversely others described the improvements POCTs offer to doctor-patient communication, for example when providing an objective test to justify why antibiotics are not required for self-limiting viral illness. Verbatim quotes to highlight the clinical barriers and facilitators to POCT adoption are provided in Text Box 1.

**Verbatim quotations to highlight the clinical barriers and facilitators to POCT adoption**
"it is a challenge to deliver equivalent analytical accuracies you can achieve within the main laboratory" (R)
"clinicians may use the tests for other conditions for which they're not evaluated" (CI)
"*there may also be kind of a tendency where doctors may rely more on the diagnostic test than on their own clinical skills*" (CI)
"I think that point of care offers the opportunity for clinicians to, if correctly used, to improve the quality of care of their patients" (I)
"Point-of-care introduces massive fundamental changes to practices" (I)

Text Box 1: **Verbatim quotations to highlight the clinical barriers and facilitators to POCT adoption (I = Industry, CI = Clinician, R = Regulator)**

For peer review only - http://bmjopen.bmj.com/site/about/guidelines.xhtml

Cultural Barriers and Facilitators

Many stakeholders felt that there still exists a lack of awareness to the POCT technology that is currently available and their potential benefits. Furthermore, a

**Verbatim quotations to highlight the cultural barriers and facilitators to POCT adoption**
"more supportive and less distrustful of point of care devices" (I)
"Doctors are the most reluctant group, in many ways, to change something, and to change a habit" (Co)
"the biggest single barrier is endorsement and support by medical clinicians" (P)
NHS and industry have to work together (I)
"I see change but I see it over a period of years and not weeks and months" (Co)

particular frustration from industry and patients was the lack of engagement with POCTs by the clinical profession. POCT technology was recognised to require a fundamental cultural change in clinical practice. Driving such change was felt to require widespread incentivisation that was usually financial with examples cited in the section relating to *Financial Barriers and Facilitators.*

Despite such challenges it was noted that enthusiasm from those with experience of POCTs was contagious, and local momentum was seen as a significant enabler. Key opinion leaders and early adoption sites were seen to actively contribute towards adoption as were industry and NHS partnerships. Verbatim quotations to highlight the cultural barriers and facilitators to POCT adoption are provided in Text Box 2.

Text Box 2: **Verbatim quotations to highlight the cultural barriers and facilitators to POCT adoption (P = Patient, I = Industry, Com = Commissioner)**

Evidence Barriers and Facilitators

For peer review only - http://bmjopen.bmj.com/site/about/guidelines.xhtml

The evidence required to facilitate POCT adoption has been described in the POCKET checklist [11] but two particular components were highlighted by stakeholders that enable adoption at a local level. Firstly, the availability of patient level data to demonstrate that the implementation of a POCT can actually make a difference to patient care within a population and secondly, local pilot studies to show that a POCT programme can actually work in a particular local practice. However, stakeholders commented that such examples were often not shared, and improvements were needed in the ability of healthcare organizations to learn from the successes of others. This was especially felt by industry stakeholders who criticised the need for repetitive local evaluations assessing impact when this had already been demonstrated in equivalent populations or health service contexts.

The challenges of effective evidence generation in POCT technology are universal to all in-vitro diagnostics and were frequently commented on; namely the cost of evaluation, the length of time high quality studies take to undertake at a time when there is a rapid rate of technological development and the methodological gaps. Verbatim quotations to highlight the evidence barriers and facilitators to POCT adoption are provided in Text Box 3.

**Verbatim quotations to highlight the evidence barriers and facilitators to POCT adoption**
"*you need a second raft of evidence that the intervention actually makes a difference to patient care or patient perception of care" (Co)*
"*we are very keen to try and get good examples of how it* [POCT strategies] *can actually work in real practice in a local environment*" (I)
"*there is no properly well established methodology like you would have for drugs*" (I)
"*that everyone in the UK wants to repeat what's already been done"* (I)

For peer review only - http://bmjopen.bmj.com/site/about/guidelines.xhtml

"*money, time to evaluate them properly, understanding where they fit in the pathway, whether it fits in with the tariff system, and I think a genuine lack of understanding of how you demonstrate health gain in a sustainable way*" (Co)

Text Box 3 Verbatim quotations to highlight the evidence barriers and facilitators to POCT adoption (I = Industry, Co = Commissioner)

Financial Barriers and Facilitators

The portrayal of POCTs as being costly was a significant barrier to their adoption. Whilst this may be "*perceived*" or a "*misperception*", the increased cost of POCT devices on a test-by-test basis caused a front-loading of cost that was unattractive to payers. The presence of budget silos within the NHS was frequently cited as a barrier particularly relevant to POCTs as there is often a mismatch between who funds the test and who saves as a consequence of implementation. Furthermore, identifying the payer within an organisation is frequently unclear, causing uncertainty within industry regarding whom to approach. This was compounded by a lack of appropriate reimbursement schemes for diagnostics, particularly in primary care, leading to a disincentive to adopt POCTs as laboratory diagnostics have already been purchased through contracted agreements with a local trust.

Challenges were highlighted in engagement with clinical commissioning groups (CCG) regarding diagnostics with the current national set up allowing for geographical variation. A particular example of this was POCT anti-coagulation testing for patients taking warfarin. Conversely, at a local CCG level POCTs were identified in contributing to establishing an increasing number of local enhanced services, for example POCT D-dimer measurement for patients with suspected deep vein thrombosis. Overall, lack of investment in new technology was a clear frustration to industry and there was awareness of the need for decision makers to recognise

For peer review only - http://bmjopen.bmj.com/site/about/guidelines.xhtml

the extensive societal cost savings that POCT investment can bring. Verbatim quotations to highlight the financial barriers and facilitators to POCT adoption are provided in Text Box 4.

For peer review only

For peer review only - http://bmjopen.bmj.com/site/about/guidelines.xhtml

Verbatim quotations to highlight the financial barriers and facilitators to POCT adoption
"*point of care testing is perceived as more expensive*" (Co)
"*an additional cost or inconvenience or a burden (…) and an extra thing they* [healthcare professionals] *have to manage*" (P)
"*it's often the barriers over the initial cost"* (R)
"*who pays for it, and who sees the reward"* (Co)
"*you have to go very high in the trust where it's almost like a triangle of where the budget lies"* (I)
"*why invest more money into putting loads of evidence behind a new technology when history shows it might not get adopted*" (I)
"*There are huge savings for the healthcare system. I think the big advantages are about patient flow and patient journeys"* (I)

Text Box 4: **Verbatim quotations to highlight the financial barriers and facilitators to POCT adoption (P = Patient, I = Industry, Co = Commissioner, R = Regulator)**

Organisational Barriers and Facilitators

The benefits of POCT technology to healthcare systems such as the NHS were clear in the interviews. They included (i) integrating POCT systems into information technology systems so that results can be shared between patients at home, primary and secondary care; (ii) improving patient flow through the availability of rapid results for decision making; and (iii) providing rural practices with access to local diagnostic services.

The relationship between clinical laboratory services and POCT sites was a contentious issue. Many stakeholders cited the laboratories as a barrier to POCT

For peer review only - http://bmjopen.bmj.com/site/about/guidelines.xhtml

adoption given the perceived threats to their workload and income. However, there was an equal cohort of participants that saw the role of clinical laboratory services as facilitating the use of POCTs by contributing their expertise in asset management, quality assurance and training. There were particular examples of good practice from Cambridgeshire and Oxfordshire of laboratories supporting the appropriate use of POCTs in the community in the same way point-of-care committees act in hospital trusts.

Other organisational barriers highlighted were the difficulty in keeping control of assets within organisations as POCTs became more available and widespread, the challenges of reconfiguring services to accommodate the increased length of consultations that incorporate POCTs and the increased workload they can bring. Hospital trust autonomy represented a challenge for industry, who described the absence of a top down adoption policy for devices in the NHS, increasing the investment required to see their devices widely adopted. National government policy was cited as a means to improve the use of POCTs in the NHS. Industry participants also felt threatened by the changing landscape for device regulation. Verbatim quotations to highlight the organisational barriers and facilitators to POCT adoption are provided in Text Box 5.

For peer review only

For peer review only - http://bmjopen.bmj.com/site/about/guidelines.xhtml

**Verbatim quotations to highlight the organisational barriers and facilitators to POCT adoption**
"the lab services look at point of care testing as being a bit of a threat." (Co)
"the laboratories are very reluctant to let go" (I)
"they are starting to work up their knowledge of how they might be able to support appropriate point of care testing in different settings, so that their mindset is changing" (Co)
"If I see a patient every ten minutes, how on earth am I going to get a point of care test in unless it delivers its results within a couple of minutes" (Cl)
"balance between how long it [POCT] takes and how many patients you could see" (Cl)
I think back in the old days when the NHS was one organisation and you could just say 'right everyone does this' then you could get away with it but I don't think we can do it anymore" (I)

Text Box 5: **Verbatim quotations to highlight the organisational barriers and facilitators to POCT adoption (I = Industry, Cl = Clinician, Co = Commissioner)**

Usability and Quality Assurance Barriers and Facilitators

Quality assurance was recognised as vital for patient safety and clinical governance but presented significant challenges to successful adoption. Highlighted barriers were the maintenance required, training for multiple users, incompatibility with local information technology systems and an absence of technical support services. Usability was closely related to quality assurance as good design was felt to reduce training requirements and make devices safer to use. Improvements in this respect, especially when providing better functionality, facilitated adoption and diffusion.

For peer review only - http://bmjopen.bmj.com/site/about/guidelines.xhtml

Verbatim quotations to highlight the design and quality assurance barriers and facilitators to POCT adoption are provided in Text Box 6.

For peer review only

For peer review only - http://bmjopen.bmj.com/site/about/guidelines.xhtml

**Verbatim quotations to highlight the usability and quality assurance barriers and facilitators to POCT adoption**
"*doctors believe that they're endowed by God to be able to perform analytical measurements without any training or need for any quality assurance unfortunately this is simply not the case*" (R)

Text Box 6: **Verbatim quotations to highlight the usability and quality assurance barriers and facilitators to POCT adoption (R = Regulator)**

Patient Factors Facilitators

Many stakeholders emphasised the popularity of POCTs to patients. POCT strategies were often seen to improve the patient journey and provide patients with a better healthcare experience. This has led to patient demand for the implementation of POCTs in many clinical pathways, often encouraged by media coverage. In the management of chronic disease the ability of POCTs to give patients control of their own healthcare was a significant benefit of the technology. All these factors were seen as enablers to the use of POCTs and no patient associated barriers were identified. Verbatim quotations to highlight the patient related facilitators to POCT adoption are summarised in Text Box 7.

**Verbatim quotations to highlight the patient factor facilitators to POCT adoption**
"*patients themselves can see the advantage of it* [POCT]" (R)
"*it's quite difficult to say no to patients when they want a test*" (Cl)
"*for me, it's been the most invigorating experience" (P)*

Text Box 7: **Verbatim quotations to highlight the patient factor facilitators to POCT adoption (P = Patient, Cl = Clinician, R = Regulator)**

For peer review only - http://bmjopen.bmj.com/site/about/guidelines.xhtml

Other (Non–Financial) Resource Use Facilitators

This final theme covers the reduction in patient-healthcare interactions that POCTs can lead to, namely a reduction in outpatient appointments, hospital admissions and length of stay. No barriers were identified relating to other (non-financial) resource use. Verbatim quotations to highlight the other (non–financial) resource use facilitators to POCT adoption are provided in Text Box 8.

**Verbatim quotations to highlight the other (non-financial) resource use facilitators to POCT adoption**
"a lot of appointments are taken up with people coming back for results, a lot of resources, text messages; if you've got the results there and then and they've got the results they don't need to phone back and people are satisfied, they go away with a printout" (Cl)
"a bigger, more strategic view, and that is this whole issue of (…) trying to keep patients out of hospital" (Com)
"*I am a patient in lots of things and I'd rather go and see my GP and then be able to say, "Well actually it's still a bit high, let's change it now," and that be done in the one visit than me going, me get a form, me have to go somewhere else to get my blood tests done*" (I)

Text Box 8: **Verbatim quotations to highlight the other (non-financial) resource use facilitators to POCT adoption (= Industry, Cl = Clinician, Com = Commissioner)**

DISCUSSION

This study has identified 32 barriers and 27 facilitators to the adoption of POCT technology. Most emergent themes contained a balance of barriers and facilitators

For peer review only - http://bmjopen.bmj.com/site/about/guidelines.xhtml

that were often closely inter-related. It is unlikely that all of these will apply to one particular device but will be dependent on factors such as the population, clinical pathway and experience of user. Many of the barriers and facilitators identified are closely related, for example the cost of the test against potential pathway cost savings. This implies that some of the barriers may be perceived rather than absolute and therefore often can be readily overcome with the right evidence, education or design modification. However, any barrier that is discovered during a device's development and seen as absolute, such as a lack of a real clinical need, should raise question as to whether there is value in continuing with development or whether a change of focus or design may be required.

Each stakeholder group has different motivations for POCT adoption. Patient care is central to all; but clinicians may be influenced by their speciality and the particular patients under their care, patients themselves respond to personal experiences, commissioners will likely have a defined focus on pathway benefits, as will regulators at a wider societal level and finally industry will be more focused on commercial aspects. These different motivations explain the variation in barriers and facilitators identified by each group. Whilst some were highlighted by most stakeholder groups (eg cost of test, siloed budgets, patient experience or training requirements) many others were more frequently picked up by one particular group such as industry's frustration at the lack of clinician buy-in or commissioner's focus on reducing attendance at healthcare services, reconfiguration of pathways and facilitating local enhanced services. Reimbursement and funding mechanisms for new POCT will be test and setting specific and examples can be seen from the ways POCT testing for C-reactive protein can be established in primary care.[5] When looking at stakeholder motivations it may be more useful to describe the barriers and facilitators as incentives or disincentives to adoption as often the implementation of a POCT strategy will bring advantages to some stakeholders but disadvantage (usually financially) others.

For peer review only - http://bmjopen.bmj.com/site/about/guidelines.xhtml

This study supports and builds on the findings of the previous literature described in the introduction with the advantage of incorporating the diverse range of stakeholders included in the present study to allow for a comprehensive presentation of adoption influencers to be reported. The two most commonly cited barriers to POCT adoption in the current study were the cost of the test and associated training burden. This directly parallels the results of the systematic review of *Quinn et al* [9] where the most commonly category retrieved articles were attributed to were economic issues and quality assurance and regulatory issues. Furthermore, when the emergent themes from this study are compared to the barriers identified in the interim report of the AAR [8] there is also good correlation. The themes from the AAR were *culture, data and evidence, finance and budgeting, systematic complexity and informational complaints, capacity/capability, dominance of the pharma paradigm and immaturity/uncertainty of landscape,* all of which were highlighted by the stakeholders we recruited in the present study. Stakeholders frequently used the evaluation of pharmaceuticals as a comparator, usually envying their established methodology and large budgets for evidence generation and marketing. This was echoed in the AAR interim report, where pharmaceutical industry was described as dominating medical technology adoption given that NICE processes are based on pharmaceutical technology, horizon scanning is limited outside of pharmaceuticals and the challenging approval pathway for companion diagnostics.

This interview study highlighted several good examples of the use of POCTs but these tended to be small-scale pilot studies involving a small local practice or population. Furthermore, these were frequently short-term projects that were discontinued once funding came to an end, regardless of level of success. Such pilot schemes were rarely published or disseminated so other regions often have to re-invent POCT pathways leading to a repetitive evaluation processes and adding to the cost of establishing POCT services. This approach can be described as ‘bottom-up’ adoption and, with one exception, no examples were provided of ‘top-down’ adoption

For peer review only - http://bmjopen.bmj.com/site/about/guidelines.xhtml

approach with central bodies driving adoption at a national level. Significant healthcare reform would be required to achieve this but ‘top-down’ adoption may be able to significantly increase the number of appropriate POCTs that are adopted following the rigorous generation of high-quality evidence of patient and service benefits.

This study has limitations. Recruitment strategy varied between stakeholder groups and aimed to provide a representative sample of participants but due to the time burden associated with conducting semi-structured interviews a degree of convenience sampling was unavoidable and the study may be prone to recruitment bias. Furthermore, participants were often providing a perspective from their experiences within the UK National Health Service and it is unclear how transferable some of the barriers and facilitators identified would relate to different healthcare systems. One further limitation relating to this study is that the interviews were designed to develop the POCKET checklist with the identification of barriers and facilitators being undertaken as a secondary objective. This led to all POCTs being grouped together and many of the barriers and facilitators may be unique to a particular POCT testing strategy (such as self testing, primary care testing etc). However, relevant questions were included in the topic guide with a diverse group of stakeholders to comprehensively identify all the barriers and facilitators they have encountered with their expertise and experience.

This study provides a comprehensive overview of the barriers and facilitators to the adoption of POCTs. In the most part these can be seen as incentives and disincentives. Widespread adoption of POCT technology can only occur if the disincentives can be predicted and mitigated. Therefore, these factors should be addressed as early as possible in the device development pathway to maximise the likelihood of widespread adoption. Qualitative research methodology including clinical needs assessments and stakeholder analyses can be used to assist this

For peer review only - http://bmjopen.bmj.com/site/about/guidelines.xhtml

process and should all be undertaken early are regularly reviewed throughout the device development pathway.

REFERENCES

1 Huddy JR, Ni MZ, Markar SR, *et al.* Point-of-care testing in the diagnosis of gastrointestinal cancers: Current technology and future directions. *World J Gastroenterol* 2015;**21**:4111. doi:10.3748/wjg.v21.i14.4111

2 Price CP. Point of care testing. *BMJ* 2001;**322**:1285–8. doi:10.1136/bmj.322.7297.1285

3 Rogers EM. *Diffusions of Innovations*. 1995.

4 Morris ZS, wooding S, Grant J. The answer is 17 years, what is the question: Understanding time lags in translational research. *J R Soc Med* Published Online First: 2011. doi:10.1258/jrsm.2011.110180

5 Huddy JR, Ni MZ, Barlow J, *et al.* Point-of-care C reactive protein for the diagnosis of lower respiratory tract infection in NHS primary care: A qualitative study of barriers and facilitators to adoption. *BMJ Open* Published Online First: 2016. doi:10.1136/bmjopen-2015-009959

6 Van Adrichem EJ, De Zande SCV, Dekker R, *et al.* Perceived barriers to and facilitators of physical activity in recipients of solid organ transplantation, a qualitative study. *PLoS One* Published Online First: 2016. doi:10.1371/journal.pone.0162725

7 Witzel TC, Rodger AJ, Burns FM, *et al.* HIV self-testing among men who have sex with men (MSM) in the UK: A qualitative study of barriers and facilitators, intervention preferences and perceived impacts. *PLoS One* Published Online First: 2016. doi:10.1371/journal.pone.0162713

8 Access A. Accelerated Access Review: Interim report. 2015.

9 Quinn AD, Dixon D, Meenan BJ. Barriers to hospital-based clinical adoption of point-of-care testing (POCT): A systematic narrative review. *Crit Rev Clin Lab*

For peer review only - http://bmjopen.bmj.com/site/about/guidelines.xhtml

Sci 2016;**53**:1–12. doi:10.3109/10408363.2015.1054984

10 Jones CH, Howick J, Roberts NW, *et al.* Primary care clinicians' attitudes towards point-of-care blood testing: a systematic review of qualitative studies. *BMC Fam Pract* 2013;**14**:117. doi:10.1186/1471-2296-14-117

11 Huddy JR, Ni M, Misra S, *et al.* Development of the Point-of-Care Key Evidence Tool (POCKET): A checklist for multi-dimensional evidence generation in point-of-care tests. *Clin Chem Lab Med* Published Online First: 2019. doi:10.1515/cclm-2018-1089

12 Tong A, Sainsbury P, Craig J. Consolidated criteria for reporting qualitative research (COREQ): A 32-item checklist for interviews and focus groups. *Int J Qual Heal Care* Published Online First: 2007. doi:10.1093/intqhc/mzm042

13 Huddy JR, Ni M, Mavroveli S, *et al.* A research protocol for developing a Point-Of-Care Key Evidence Tool 'POCKET': a checklist for multidimensional evidence reporting on point-of-care in vitro diagnostics: Figure 1. *BMJ Open* 2015;**5**:e007840. doi:10.1136/bmjopen-2015-007840

14 Tong A, Sainsbury P, Craig J. Consolidated criteria for reporting qualitative research (COREQ): a 32-item checklist for interviews and focus groups. *Int J Qual Health Care* 2007;**19**:349–57. doi:10.1093/intqhc/mzm042

AUTHORS CONTRIBUTIONS

GBH, JRH and JB conceived the project. GBH, JRH, JB and MN planned the project. JRH undertook the data collection. JRH and MN analysed the data. GBH, JRH, JB, and MN were involved in interpreting the data. All authors were involved in drafting the final manuscript.

For peer review only

TABLES

Theme	Identified Barrier	Comm.	Clin.	Reg.	Industry	Patients	Total
**Clinical**	Inappropriate use of test	1	1	1	0	1	**4**
	Lack of clinical utility	1	4	0	2	0	**7**
	Reduced accuracy	0	2	2	0	0	**4**
	Discourages use of clinical judgement	0	1	0	1	0	**2**
**Cultural**	Lack of awareness	0	0	0	2	0	**2**
	Lack of clinician buy-in	0	0	0	6	1	**7**
	Resistance to change	4	0	1	2	0	**7**
	Need for service reconfiguration	5	0	0	2	0	**7**
	Lack of Accountability	0	0	0	0	1	**1**
**Evidence**	Cost of evidence generation	0	1	0	2	0	**3**
	Lack of evidence	2	0	1	0	0	**3**
	Lack of evaluation methodology	0	1	0	1	0	**2**
	Repetition of small scale local studies	0	0	0	3	0	**3**
	Rate of technology development	0	0	1	0	0	**1**

For peer review only - http://bmjopen.bmj.com/site/about/guidelines.xhtml

**Financial**	Commissioning	1	0	0	0	3	**4**
	Cost of test	5	3	1	3	2	**14**
	Lack of investment in new technology	0	1	0	3	0	**4**
	Payer identification	1	0	0	2	0	**3**
	Reimbursement	0	0	2	0	0	**2**
	Siloed budgets	1	1	1	1	0	**4**
**Organisational**	Asset control	0	0	1	0	0	**1**
	Duration of consultation	1	2	1	0	0	**4**
	Increased workload	2	1	0	1	0	**3**
	Threat to laboratory services	4	0	1	3	0	**8**
	Reduced throughput	0	0	1	0	0	**1**
	Regulation	0	0	0	2	0	**2**
	Trust Autonomy	0	0	0	2	0	**2**
**Design and Quality Assurance**	Maintenance Requirements	1	0	1	0	0	**2**
	Training Burden	2	6	4	2	0	**14**
	Poor Usability	0	0	1	1	0	**2**

For peer review only

	Lack of connectivity	1	0	0	1	0	**2**
	Lack of technical support	0	0	1	0	0	**1**

Table 1: **Identified barriers to the adoption of POCT and frequencies of stakeholders who highlighted each by stakeholder group (Comm – commissioners, Clin – clinicians, Reg - Regulators)**

For peer review only

Theme	Identified Facilitator	Comm.	Clin.	Reg.	Industry	Patients	Total
Clinical	Improving doctor-patient communication	2	0	0	0	0	**2**
	Improving patient care	0	2	1	2	0	**5**
	Time to result	0	4	1	2	0	**7**
	More effective triage	0	0	0	1	0	**1**
	Reduced time to treatment	4	0	5	1	0	**10**
Cultural	Word of mouth	0	0	0	0	4	**4**
	Incentives to adopt	0	1	0	1	0	**2**
	Key opinion leaders	1	0	0	0	2	**3**
	NHS and industry partnerships	1	1	0	1	0	**3**
Evidence	Availability of patient level data	1	0	1	0	0	**2**
	Successful pilot data (local level)	0	0	0	2	0	**2**
Financial	Cost savings	1	0	1	1	0	**3**
	Improved efficiency	3	2	1	2	0	**8**
Organisational	Connectivity	0	0	0	0	2	**2**
	Flow	0	0	0	0	2	**2**

For peer review only

	Government policy	0	0	0	1	0	**1**
	POCT use in local enhanced services	3	0	0	0	0	**3**
	Locality of diagnostics	0	2	0	2	0	**4**
	Support from central pathology laboratories	2	1	1	1	0	**5**
**Design and Quality Assurance**	Good design and functionality	0	1	0	1	0	**2**
	Usability	1	0	1	0	0	**2**
**Patient Factors**	Patient demand	0	1	1	0	0	**2**
	Patient experience/patient journey	3	1	2	2	0	**8**
	Patient ownership	3	0	1	1	7	**12**
**Resource Use**	Reduced attendance to healthcare services	6	1	1	0	0	**8**
	Reduced hospital admissions	0	0	1	1	0	**2**
	Reduced length of stay	0	0	1	1	0	**2**

Table 2 Identified facilitators to the adoption of POCT and frequencies of stakeholders who highlighted each by stakeholder group (Comm – commissioners, Clin – clinicians, Reg - Regulators)

For peer review only

For peer review only - http://bmjopen.bmj.com/site/about/guidelines.xhtml

For peer review only

For peer review only - http://bmjopen.bmj.com/site/about/guidelines.xhtml

APPENDIX

Appendix 1: POCKET Interview prompt (initial version)

Section 1: Point-of-Care Tests

Clinical Needs Assessment

Example Introductory Statement
"Point-of–care diagnostic tests allow for diagnostic tests to be performed at the bedside, in the clinic or in the patient's home. With advancing diagnostic technologies new point of care tests can give rapid or instant results allowing for real-time clinical decision making in many diseases."

1. What is your opinion of point-of-care diagnostic tests?

   Prompt if required for advantages/disadvantages

2. Where are they required? Why?

3. How can we know where POC diagnostic tests would give most benefit?

   a. (clinical needs assessment/unmet clinical need) Explore unmet needs.

4. Please mark which of the following are useful to determine unmet clinical needs:
   a. Systematic literature reviews
   b. Expert opinion
   c. Patient opinion
   d. Competition analysis
   e. Other

5. Who should be establishing unmet needs?

Section 2: Evidence and Evaluation

1. Once a new point of care diagnostic test has been produced what should the next steps be?

   Prompts: How should we evaluate these devices? Why?

2. What are the goals of evidence generation in new point-of-care diagnostic tests?

1. What evidence best leads to adoption?

2. Do you have any examples of devices that have been adopted easily or with particular difficulty. What caused this? Prompt: home coagulation checking.

3. Please rank the evidence you regard as vital, useful, not required or unsure for the implementation of point of care devices that will be used **in healthcare institutions**?

For peer review only

a. Clinical trials
b. Usability assessment
c. Design Evaluation
d. Patient experience
e. Clinical needs assessment
f. Clinical Pathway Mapping
g. Patient safety/Risk Assessment
h. Economic Analysis
i. Stakeholder Analysis
j. Barriers to adoption
k. Decision Analysis
l. Other

4. Please rank the evidence you regard as vital, useful, not required or unsure for the implementation of point of care devices that will be used **in patient's homes**?

   a. Clinical trials
   b. Usability assessment
   c. Design Evaluation
   d. Patient experience
   e. Clinical needs assessment
   f. Clinical Pathway Mapping
   g. Patient safety/Risk Assessment
   h. Economic Analysis
   i. Stakeholder Analysis
   j. Barriers to adoption
   k. Decision Analysis
   l. Other

5. Where should this evidence come from?
   a. Industry
   b. Academia
   c. Industry/academia partnerships
   d. Consultancy firms
   e. Other

6. What incentives can enhance the adoption of point-of-care test devices?

<u>Validity</u>

Example Introductory Statement
"We will now discuss clinical validity or the accuracy of a diagnostic test."

1. What should evidence on clinical validity demonstrate?

2. Please rank the usefulness of these sources of evidence for clinical validity?

   a. Randomised controlled trial evidence
   b. Diagnostic Accuracy Studies (Case-control)
   c. Linked Evidence Approaches
   d. Bayesian Networks
   e. Other approach

For peer review only

3. What if there is no reference standard?

"Point-of-care tests are often seen as a disruptive innovation, by which I mean that the introduction of the test disturbs the established clinical pathway. This can be demonstrated by clinical pathway modeling to predict the impact of implementation."

4. Should new tests be modeled against current practice or best practice?

5. How translatable is this evidence between different institutions or different healthcare systems?

6. Is a trade off in accuracy acceptable for the convenience of point-of-care tests?

7. Any other thoughts regarding the accuracy or validity of POC tests?

Usability

Introductory Statement
"Point-of-care tests are often performed away from central laboratories or in the patient's home and therefore trained laboratory personnel are not present and those using the test will have limited training. Therefore, these devices need to be user-friendly."

1. What are your thoughts on the usability of point-of care diagnostic test?

2. Should this be tested? How?

3. Is evidence demonstrating the usability of a device important for implementation?

4. How should this be presented?

5. What usability assessments are you aware of?

6. Is evidence on usability required that is over and above what is required for regulatory approval (FDA/CE marking)?

7. How does patient safety impact on implementation of new tests?

    a. Prompt if required re risk assessment

8. Any other thoughts on the usability assessment of point-of-care diagnostic tests?

Economic Analysis

Introductory Statement
"Healthcare budgets are a finite resource and cost-effectiveness needs to be demonstrated to justify the use of new diagnostic tests."

For peer review only

1. What do you think gives value to new point-of-care diagnostic tests?

2. How is this measured?

3. What economic information would benefit the implementation of point-of-care diagnostic tests?

4. Are modeling techniques reliable? Why?

7. Are quality adjusted life years (QALY) a useful and sufficient measurement of value in the evaluation of diagnostic tests?

8. Any other thoughts regarding the economic analysis of point-of-care diagnostic tests?

<u>Patient Experience</u>

Introductory statement
"Patients are the reason why diagnostic tests are performed so when engaging with patients regarding new technologies…"

1. How should patient experience and opinion be incorporated into the evaluation of new point-of-care diagnostic tests?

2. What evidence should patients have in new diagnostic tests?

<u>Section 3: Adoption</u>

<u>Barriers</u>

Example Introductory statement
"Even with good evidence some point-of care-technologies fail or are slow to be implemented."

1. What are the challenges in generating evidence to justify the implementation of new point-of-care diagnostic tests?

1. How can these be overcome?

2. Is there any other evidence you feel would assist in the implementation of new point-of-care diagnostic tests?

3. Are there any other barriers to the implementation of new point-of-care diagnostic tests?

4. What can the NIHR Diagnostic Evidence Co-operatives do to help you make decisions?

5. NICE/Industry/CCG: As a partner to the Diagnostic Evidence Co-operatives, how do you envisage us working together?

<u>Efficient Evidence Generation</u>

For peer review only

Example Introductory Statement
"Effective study design may allow evidence in differing domains to be gathered more efficiently."

1. How can we be more efficient in the evaluation process of point-of-care diagnostic tests?

2. What changes would you like to see?

3. Are you aware of Bayesian Networks being used to combine sources of evidence?

4. Any other thoughts or comments regarding point-of-care tests and evidence generation?

For peer review only

COREQ checklist for semi-structured interview checklist [14]

Domain 1: Research team and reflexivity			
Personal Characteristics			
1	Interviewer/facilitator	Which author/s conducted the interview or focus group?	JRH
2	Credentials	What were the researcher's credentials? E.g. PhD, MD	BMedSci BMBS MRCS
3	Occupation	What was their occupation at the time of the study?	Clinical Research Fellow
4	Gender	Was the researcher male or female?	Male
5	Experience and training	What experience or training did the researcher have?	This was the first qualitative study undertaken by the researcher. A co-researcher with experience in qualitative research supported the work and the primary researcher undertook a course in qualitative research methodology during the study.
Relationship with participants			
6	Relationship established	Was a relationship established prior to study commencement?	There was no prior relationship with 38/43 participants. The researcher through previous academic collaboration knew five participants.
7	Participant knowledge of the interviewer	What did the participants know about the researcher? e.g. personal goals, reasons for doing the research	Participants knew that the work was being undertaken by a clinical research fellow towards a PhD under the remit of the NIHR Diagnostic Evidence Co-operative. Participants knew the overall aims of the project.
8	Interviewer characteristics	What characteristics were reported about the interviewer/facilitator? e.g. Bias, assumptions, reasons and interests in the research topic	As above
Domain 2: study design			
Theoretical framework			

For peer review only

9	Methodological orientation and Theory	What methodological orientation was stated to underpin the study? e.g. grounded theory, discourse analysis, ethnography, phenomenology, content analysis	This study was based on grounded theory.
Participant selection			
10	Sampling	How were participants selected? e.g. purposive, convenience, consecutive, snowball	Sampling was dependent on stakeholder group. Purposive sampling was intended but convenience sampling undertaken when recruitment did not allow.
11	Method of approach	How were participants approached? e.g. face-to-face, telephone, mail, email	Email
12	Sample size	How many participants were in the study?	43 participants were included in total with 8-10 in each stakeholder group.
13	Non-participation	How many people refused to participate or dropped out? Reasons?	This is not known for the patients, industry and regulators group as participants were approached by a body external to the research group. Two invited clinicians did not participate (one did not reply to invitation and one did not have mutual availability for interview). Twenty commissioners declined to participate (two declined as did not have experience of subject and 18 did not reply to invitation).
Setting			
14	Setting of data collection	Where was the data collected? e.g. home, clinic, workplace	Workplace. The exception to this was patients were data was collected by telephone interview with the patient at home.
15	Presence of non-participants	Was anyone else present besides the participants and researchers?	No, with the exception of the two interviews that were undertaken with two participants.
16	Description of sample	What are the important characteristics of the sample? e.g. demographic data, date	Available data provided in study report
Data collection			
17	Interview guide	Were questions, prompts, guides provided by the authors? Was it pilot tested?	Yes (see appendix).
18	Repeat interviews	Were repeat interviews carried out? If yes, how many?	No

For peer review only

19	Audio/visual recording	Did the research use audio or visual recording to collect the data?	Audio recording
20	Field notes	Were field notes made during and/or after the interview or focus group?	No
21	Duration	What was the duration of the interviews or focus group?	Median length of 31 minutes and 21 seconds (range 11 minutes and 50 seconds - 58 minutes and 20 seconds)
22	Data saturation	Was data saturation discussed?	Yes
23	Transcripts returned	Were transcripts returned to participants for comment and/or correction?	No, the methodology allowed for clarification of comments but this was not necessary during analysis.
Domain 3: analysis and findings			
Data analysis			
24	Number of data coders	How many data coders coded the data?	Two
25	Description of the coding tree	Did authors provide a description of the coding tree?	Yes
26	Derivation of themes	Were themes identified in advance or derived from the data?	Some themes were identified in advance but these were modified during the analysis process.
27	Software	What software, if applicable, was used to manage the data?	NVivo
28	Participant checking	Did participants provide feedback on the findings?	No
Reporting			
29	Quotations presented	Were participant quotations presented to illustrate the themes / findings?<br><br>Was each quotation identified? e.g. participant number	Yes<br><br>Yes – stakeholder group identified
30	Data and findings consistent	Was there consistency between the data presented and the findings?	Yes
31	Clarity of major themes	Were major themes clearly presented in the findings?	Yes
32	Clarity of minor themes	Is there a description of diverse cases or discussion of minor themes?	Yes

For peer review only

For peer review only

BMJ Open

A Qualitative Analysis of Stakeholder Interviews to Identify the Barriers and Facilitators to the Adoption of Point-of-Care Diagnostic Tests in the United Kingdom

Journal:	*BMJ Open*
Manuscript ID	bmjopen-2020-042944.R1
Article Type:	Original research
Date Submitted by the Author:	21-Jan-2021
Complete List of Authors:	Huddy, Jeremy; Imperial College London, Surgery and Cancer<br>Ni, Melody; Imperial College London, Surgery and cancer<br>Barlow, James; Imperial College London, Business School<br>Hanna, George; Imperial College London, Faculty of Medicine, Department of Surgery & Cancer
**Primary Subject Heading**:	Diagnostics
Secondary Subject Heading:	Health policy
Keywords:	Health economics < HEALTH SERVICES ADMINISTRATION & MANAGEMENT, Health policy < HEALTH SERVICES ADMINISTRATION & MANAGEMENT, Clinical chemistry < PATHOLOGY

SCHOLARONE™
Manuscripts

I, the Submitting Author has the right to grant and does grant on behalf of all authors of the Work (as defined in the below author licence), an exclusive licence and/or a non-exclusive licence for contributions from authors who are: i) UK Crown employees; ii) where BMJ has agreed a CC-BY licence shall apply, and/or iii) in accordance with the terms applicable for US Federal Government officers or employees acting as part of their official duties; on a worldwide, perpetual, irrevocable, royalty-free basis to BMJ Publishing Group Ltd ("BMJ") its licensees and where the relevant Journal is co-owned by BMJ to the co-owners of the Journal, to publish the Work in this journal and any other BMJ products and to exploit all rights, as set out in our licence.

The Submitting Author accepts and understands that any supply made under these terms is made by BMJ to the Submitting Author unless you are acting as an employee on behalf of your employer or a postgraduate student of an affiliated institution which is paying any applicable article publishing charge ("APC") for Open Access articles. Where the Submitting Author wishes to make the Work available on an Open Access basis (and intends to pay the relevant APC), the terms of reuse of such Open Access shall be governed by a Creative Commons licence – details of these licences and which Creative Commons licence will apply to this Work are set out in our licence referred to above.

Other than as permitted in any relevant BMJ Author's Self Archiving Policies, I confirm this Work has not been accepted for publication elsewhere, is not being considered for publication elsewhere and does not duplicate material already published. I confirm all authors consent to publication of this Work and authorise the granting of this licence.

For peer review only

A Qualitative Analysis of Stakeholder Interviews to Identify the Barriers and Facilitators to the Adoption of Point-of-Care Diagnostic Tests in the United Kingdom

Jeremy R Huddy (PhD FRCS),[1] Melody Ni (PhD),[1] James Barlow (PhD),[2] George B Hanna (PhD, FRCS)[1]

[1]Department of Surgery and Cancer, Imperial College, London, UK

[2]Imperial College Business School, South Kensington, London, UK

The National Institute for Health Research Diagnostic Evidence Co-operative London at Imperial College Healthcare NHS Trust

St Mary's Hospital, South Wharf Road, London, W2 1NY, United Kingdom

Correspondence to:
Professor George B Hanna
Department of Surgery and Cancer
Imperial College London
Academic Surgical Unit, 10th Floor, QEQM Building
St Mary's Hospital, South Wharf Road
London, W2 1NY, United Kingdom
Tel: +44 203 312 2124
Fax: +44 203 312 6309
Email: g.hanna@imperial.ac.uk

Article category: Research Article

Word Count: 4925 words

Abstract: 215 words

Tables: 2

For peer review only - http://bmjopen.bmj.com/site/about/guidelines.xhtml

Figures: 8 text boxes

Keywords: Point-of-Care Testing

Patient and Public Involvement: The National Institute for Health Research (NIHR) Diagnostic Evidence Co-operative London at Imperial College Healthcare NHS Trust has an active patient and public involvement group who inform the identification, prioritisation, design, and execution of our research (https://london.ivd.nihr.ac.uk/patient-public/).

.

For peer review only - http://bmjopen.bmj.com/site/about/guidelines.xhtml

ABSTRACT

Objectives: This study investigated the barriers and facilitators to the adoption of point-of-care tests (POCTs).

Design: Qualitative study incorporating a constant comparative analysis of stakeholder responses to a series of interviews undertaken to design the Point-of-Care Key Evidence Tool (POCKET).

Setting: The study was conducted in relation to POCTs used in all aspects of healthcare.

Participants: Forty-three stakeholders were interviewed including clinicians (incorporating laboratory staff and members of trust POCT committees), commissioners, industry, regulators and patients.

Results: Thematic analysis highlighted 32 barriers in six themes and 28 facilitators in eight themes to the adoption of POCTs. Six themes were common to both barriers and facilitators (clinical, cultural, evidence, design and quality assurance, financial and organisational) and two themes contained facilitators alone (patient factors and other (non-financial) resource use).

Conclusions: Findings from this study demonstrate the complex motivations of stakeholders in the adoption of POCT. Most themes were common to both barriers and facilitators suggesting that good device design, stakeholder engagement and appropriate evidence provision can increase the likelihood of a POCT device adoption. However, it is important to realise that whilst the majority of identified barriers may be perceived or mitigated some may be absolute and if identified early in device development further investment should be carefully considered.

For peer review only

For peer review only - http://bmjopen.bmj.com/site/about/guidelines.xhtml

STRENGTHS AND LIMITATIONS

- Semi-structured interview study incorporated a diverse range of views from stakeholders involved in the implementation of POCT
- Thematic analysis identified the barriers and facilitators that should be considered when developing POCT
- Interview topic guide grouped all POCT together and incentives and disincentives may vary between different POCT categories (eg. patient self-testing, testing in primary care etc.)

INTRODUCTION

Point-of-care tests (POCT) are tests that are carried out in close proximity to patients by non-laboratory trained personnel either in healthcare settings or the home. Technological advances have led to more tests traditionally performed in clinical laboratories being available to clinicians at the bedside.[1] This convenience can allow for real time decision-making within patient care pathways and has led to improved access to diagnostics, reduced time to treatments, patient satisfaction and cost savings.[2] Further advantages include more accurate clinical decision making regarding when to initiate treatments and improved guidance for patients regarding when to seek advice or visit a healthcare professional.

Healthcare systems represent significant challenges to technology adoption. It is often not failures in the technology but cultural, organisational and financial aspects of the system that prevent implementation. The diffusion of innovation traditionally follows an S-shaped curve as an innovation passes through stages of adoption and spread before plateauing at mainstream adoption.[3] However, the length of time taken innovations to be adopted in health systems has been well documented around the world; one review suggests an average of 17 years.[4] Efforts need to be made to speed up this process and identify factors that can hamper or accelerate the adoption journey so that the benefits to patients and society of these devices can be realised.

For peer review only - http://bmjopen.bmj.com/site/about/guidelines.xhtml

Barriers and facilitators can be reliably studied using qualitative techniques.[5–7] A better understanding of the adoption motivations for POCTs may inform strategies to mitigate barriers and promote facilitators that expediate the time it takes for devices to become integrated into patient pathways. Barriers and facilitators to healthcare technologies were investigated as part of the 2016 Accelerated Access Review (AAR) commissioned by the UK government and supported by the Wellcome Trust).[8] This review began with an interim report that highlighted a list of barriers that stand in the way of innovation adoption in healthcare systems. However, the findings from this report reflect a wide variety of healthcare technologies including diagnostics, medical devices and pharmaceuticals findings and therefore may not represent the specific challenges of expediating the development and adoption of POCT technology. Given the increasing number of POCTs available and their potential impact across clinical pathways there is value in investigating the specific barriers and facilitators to POCT technology adoption. This would allow better understanding of the potential challenges in commercialising and implementing POCT tests, so that modifications may be made early in their development and guide the collection of evidence needed to address the needs of clinical and other stakeholders. Two systematic reviews have previously investigated such barriers; in their systematic narrative review of the barriers to hospital-based adoption of POCT Quinn et al. identified the most prevalent barrier categories being the economics of adoption, quality assurance and regulatory issues, device performance and data management and staff and operation issues.[9] Jones et al. investigated the attitudes of primary care clinicians in Europe and Australia towards POCTs and reported the three main barriers being related to the impact on clinical practice, impact on patient-clinician relationships and perceived patient experience.[10]

Previous work has led to the development of the Point-of-Care Key Evidence Tool (POCKET) tool [11] that reports the evidence that is a required by different stakeholder groups to facilitate good decision making in regards the adoption of POCT. This has

For peer review only - http://bmjopen.bmj.com/site/about/guidelines.xhtml

demonstrated that the evidence is multi-dimensional and includes evaluation of both the test and the test device itself. Currently there exists a shortfall in available translational evidence compared to what is required by decision makers [11]. As part of this study a series of in-depth semi-structured interviews was undertaken with a broad range of relevant stakeholders. The aim of this study was to undertake a qualitative analysis of these interviews with a specific focus on identifying the barriers and facilitators that exist to the adoption of POCTs within patient pathways.

METHODS

A qualitative analysis of the 41 semi-structured stakeholder interviews that were conducted to develop POCKET was undertaken using grounded theory. Two interviews were undertaken with two participants concurrently at the participant's request. The study description and results are summarised in accordance with the Consolidated Criteria for Reporting Qualitative Research (COREQ) checklist.[12] Stakeholders included were clinicians (including laboratory staff and members of trust POCT committees), commissioners, industry, regulators and patients. The methodology for the development of the POCKET checklist including the interview phase, topic guide and recruitment strategy has been previously published.[11,13] The protocol was approved by the Joint Research Compliance Office Imperial College, London, and the Imperial College Hospitals National Health Service (NHS) Trust Research and Development Department (ICREC References 14IC2186 and 14SM2190). Written consent was obtained from all participants. The interviews were undertaken by JRH. The interview participants received information relating to the study in advance of the interview through a participant information leaflet. The interviews were semi-structured and a topic list was prepared in advance and piloted (appendix 1). Interviews were tailored to the role and level of experience of the interviewee. This ensured the interviews remained relevant to the different stakeholders. The interview topic guide included a section relating to adoption where

For peer review only - http://bmjopen.bmj.com/site/about/guidelines.xhtml

barriers and facilitators were explored. Interviews were undertaken either in person, by telephone or through Skype (Microsoft, Seattle, WA, USA). Interviews were recorded. Following verbatim transcription transcripts were not returned to participants for verification. Interviews were analysed by two researchers (JRH and a clinical research nurse working within the NIHR Diagnostic Evidence Co-operative London) who independently reviewed the transcripts using a constant comparative technique before meeting to compare emergent themes. Interviews were undertaken until saturation had been reached, as demonstrated by the absence of new themes emerging from the analysis. NVivo (version 11.2.2 QSR International) was used to query the data and provide matrix coding so that the frequency of stakeholders in each group who identified a particular barrier or facilitator could be determined.

RESULTS

Forty three stakeholders comprising regulators (10), Industry (9), commissioners (8), clinicians (8) and patients (8), were interviewed between August 2014 and December 2015. Recruitment rates from invitation were 80% for clinicians and 29% for commissioners. Regulators, industry representatives and patients were recruited

through bodies external to the study group and recruitment rate is not known. Twenty-three (55%) interviews were undertaken face to face and 19 (45%) by Skype or telephone. Interviews had a median length of 31 minutes (range 12-58 minutes). For the purpose of reporting interviewees are referred to as stakeholders.

Thematic analysis highlighted 32 barriers in six themes and 28 facilitators in eight themes to the adoption of POCTs. Six themes were common to both barriers and facilitators (clinical, cultural, evidence, design and quality assurance, financial and organisational) and two themes contained facilitators alone (patient factors and other (non-financial) resource use). Tables 1 and 2 summarise the barriers and facilitators identified within each theme and the number of participants from each stakeholder

For peer review only

group who identified each. The sections below qualitatively summarise the themes that emerged during the interviews along with verbatim quotations.

Clinical Barriers and Facilitators

The benefits that POCTs are able to bring to clinical pathways were widely recognised by all stakeholders. Particular focus was given to the communication benefits, better patient care, improved turnaround time and effective patient triage. However, there was an appreciation that technological advances may have led to some POCTs being available without or before a clinical need or pathway benefit could be clearly identified. Furthermore, even when a clinical need was determined, given the volume and accuracy offered if a laboratory was able to deliver a test result at a time point that doesn't affect the efficacy of the associated intervention, then the POCT's utility can still be questioned (unless other benefits can be demonstrated). Some stakeholders were concerned by the inappropriate use of POCTs, for example patients self-testing too often or when not indicated by clinicians. POCTs were seen by some to discourage the use of clinical judgement, leading to an overreliance on guidelines and treatment algorithms; conversely others described the improvements POCTs offer to doctor-patient communication, for example when providing an objective test to justify why antibiotics are not required for self-limiting viral illness. Verbatim quotes to highlight the clinical barriers and facilitators to POCT adoption are provided in Text Box 1.

**Verbatim quotations to highlight the clinical barriers and facilitators to POCT adoption**
"it is a challenge to deliver equivalent analytical accuracies you can achieve within the main laboratory" (R)
"clinicians may use the tests for other conditions for which they're not evaluated" (CI)
"*there may also be kind of a tendency where doctors may rely more on the diagnostic test than on their own clinical skills*" (CI)
"I think that point of care offers the opportunity for clinicians to, if correctly used, to improve the quality of care of their patients" (I)

For peer review only - http://bmjopen.bmj.com/site/about/guidelines.xhtml

"Point-of-care introduces massive fundamental changes to practices" (I)

Text Box 1: **Verbatim quotations to highlight the clinical barriers and facilitators to POCT adoption (I = Industry, CI = Clinician, R = Regulator)**

**Verbatim quotations to highlight the cultural barriers and facilitators to POCT adoption**
"more supportive and less distrustful of point of care devices" (I)
"Doctors are the most reluctant group, in many ways, to change something, and to change a habit" (Co)
"the biggest single barrier is endorsement and support by medical clinicians" (P)
NHS and industry have to work together (I)
"I see change but I see it over a period of years and not weeks and months" (Co)

Cultural Barriers and Facilitators

Many stakeholders felt that there still exists a lack of awareness to the POCT technology that is currently available and their potential benefits. Furthermore, a particular frustration from industry and patients was the lack of engagement with POCTs by the clinical profession. POCT technology was recognised to require a fundamental cultural change in clinical practice. Driving such change was felt to require widespread incentivisation that was usually financial with examples cited in the section relating to *Financial Barriers and Facilitators.*

Despite such challenges it was noted that enthusiasm from those with experience of POCTs was contagious, and local momentum was seen as a significant enabler. Key opinion leaders and early adoption sites were seen to actively contribute towards adoption as were industry and NHS partnerships. Verbatim quotations to highlight the cultural barriers and facilitators to POCT adoption are provided in Text Box 2.

For peer review only - http://bmjopen.bmj.com/site/about/guidelines.xhtml

Text Box 2: **Verbatim quotations to highlight the cultural barriers and facilitators to POCT adoption (P = Patient, I = Industry, Com = Commissioner)**

Evidence Barriers and Facilitators

The evidence required to facilitate POCT adoption has been described in the POCKET checklist [11] but two particular components were highlighted by stakeholders that enable adoption at a local level. Firstly, the availability of patient level data to demonstrate that the implementation of a POCT can actually make a difference to patient care within a population and secondly, local pilot studies to show that a POCT programme can actually work in a particular local practice. However, stakeholders commented that such examples were often not shared, and improvements were needed in the ability of healthcare organizations to learn from the successes, and failures, of others. This was especially felt by industry stakeholders who criticised the need for repetitive local evaluations assessing impact when this had already been demonstrated in equivalent populations or health service contexts.

The challenges of effective evidence generation in POCT technology are universal to all in-vitro diagnostics and were frequently commented on; namely the cost of evaluation, the length of time high quality studies take to undertake at a time when there is a rapid rate of technological development and the methodological gaps. Verbatim quotations to highlight the evidence barriers and facilitators to POCT adoption are provided in Text Box 3.

For peer review only - http://bmjopen.bmj.com/site/about/guidelines.xhtml

Verbatim quotations to highlight the evidence barriers and facilitators to POCT adoption
"*you need a second raft of evidence that the intervention actually makes a difference to patient care or patient perception of care" (Co)*
"*we are very keen to try and get good examples of how it* [POCT strategies] *can actually work in real practice in a local environment*" (I)
"*there is no properly well established methodology like you would have for drugs*" (I)
"*that everyone in the UK wants to repeat what's already been done"* (I)
"*money, time to evaluate them properly, understanding where they fit in the pathway, whether it fits in with the tariff system, and I think a genuine lack of understanding of how you demonstrate health gain in a sustainable way*" (Co)

Text Box 3 Verbatim quotations to highlight the evidence barriers and facilitators to POCT adoption (I = Industry, Co = Commissioner)

Financial Barriers and Facilitators

The portrayal of POCTs as being costly was a significant barrier to their adoption. Whilst, as one commissioner described, this may be "perceived", the increased cost of POCT devices on a test-by-test basis caused a front-loading of cost that was unattractive to payers. The presence of budget silos within the NHS was frequently cited as a barrier particularly relevant to POCTs as there is often a mismatch between who funds the test and who saves as a consequence of implementation. Furthermore, identifying the payer within an organisation is frequently unclear, causing uncertainty within industry regarding whom to approach. This was compounded by a lack of appropriate reimbursement schemes for diagnostics, particularly in primary care, leading to a disincentive to adopt POCTs as laboratory diagnostics have already been purchased through contracted agreements with a local trust.

For peer review only - http://bmjopen.bmj.com/site/about/guidelines.xhtml

Challenges were highlighted in engagement with clinical commissioning groups (CCG) regarding diagnostics with the current national set up allowing for geographical variation. A particular example of this was POCT anti-coagulation testing for patients taking warfarin. Conversely, at a local CCG level POCTs were identified in contributing to establishing an increasing number of local enhanced services, for example POCT D-dimer measurement for patients with suspected deep vein thrombosis. Overall, lack of investment in new technology was a clear frustration to industry and there was awareness of the need for decision makers to recognise the extensive societal cost savings that POCT investment can bring, such as reducing the spread of infectious diseases [14]. Verbatim quotations to highlight the financial barriers and facilitators to POCT adoption are provided in Text Box 4.

For peer review only - http://bmjopen.bmj.com/site/about/guidelines.xhtml

**Verbatim quotations to highlight the financial barriers and facilitators to POCT adoption**
"*point of care testing is perceived as more expensive*" (Co)
"*an additional cost or inconvenience or a burden (…) and an extra thing they* [healthcare professionals] *have to manage*" (P)
"*it's often the barriers over the initial cost*" (R)
"*who pays for it, and who sees the reward*" (Co)
"*you have to go very high in the trust where it's almost like a triangle of where the budget lies*" (I)
"*why invest more money into putting loads of evidence behind a new technology when history shows it might not get adopted*" (I)
"*There are huge savings for the healthcare system. I think the big advantages are about patient flow and patient journeys*" (I)

Text Box 4: **Verbatim quotations to highlight the financial barriers and facilitators to POCT adoption (P = Patient, I = Industry, Co = Commissioner, R = Regulator)**

Organisational Barriers and Facilitators

The benefits of POCT technology to healthcare systems such as the NHS were clear in the interviews. They included (i) integrating POCT systems into information technology systems so that results can be shared between patients at home, primary and secondary care; (ii) improving patient flow through the availability of rapid results for decision making; and (iii) providing rural practices with access to local diagnostic services.

The relationship between clinical laboratory services and POCT sites was a contentious issue. Many stakeholders cited the laboratories as a barrier to POCT

For peer review only - http://bmjopen.bmj.com/site/about/guidelines.xhtml

adoption given the perceived threats to their workload and income. However, there was an equal cohort of participants that saw the role of clinical laboratory services as facilitating the use of POCTs by contributing their expertise in asset management, quality assurance and training. There were particular examples of good practice from Cambridgeshire and Oxfordshire of laboratories supporting the appropriate use of POCTs in the community in the same way point-of-care committees act in hospital trusts.

Other organisational barriers highlighted were the difficulty in keeping control of assets within organisations as POCTs became more available and widespread, the challenges of reconfiguring services to accommodate the increased length of consultations that incorporate POCTs and the increased workload they can bring. Hospital trust autonomy represented a challenge for industry, who described the absence of a top down adoption policy for devices in the NHS, increasing the investment required to see their devices widely adopted. National government policy was cited as a means to improve the use of POCTs in the NHS. Industry participants also felt threatened by the changing landscape for device regulation including new European Union Regulations for medical devices and in vitro diagnostic medical devices [15]. Since the interview study was undertaken these concerns are likely to have been exacerbated by the added uncertainty caused by the ongoing negotiations for the United Kingdom leaving the European Union. Verbatim quotations to highlight the organisational barriers and facilitators to POCT adoption are provided in Text Box 5.

For peer review only

For peer review only - http://bmjopen.bmj.com/site/about/guidelines.xhtml

**Verbatim quotations to highlight the organisational barriers and facilitators to POCT adoption**
"the lab services look at point of care testing as being a bit of a threat." (Co)
"the laboratories are very reluctant to let go" (I)
"*they are starting to work up their knowledge of how they might be able to support appropriate point of care testing in different settings, so that their mindset is changing"* (Co)
"If I see a patient every ten minutes, how on earth am I going to get a point of care test in unless it delivers its results within a couple of minutes" (Cl)
"balance between how long it [POCT] takes and how many patients you could see" (Cl)
I think back in the old days when the NHS was one organisation and you could just say 'right everyone does this' then you could get away with it but I don't think we can do it anymore" (I)

Text Box 5: **Verbatim quotations to highlight the organisational barriers and facilitators to POCT adoption (I = Industry, Cl = Clinician, Co = Commissioner)**

Usability and Quality Assurance Barriers and Facilitators

Quality assurance was recognised as vital for patient safety and clinical governance but presented significant challenges to successful adoption. Highlighted barriers were the maintenance required, training for multiple users, incompatibility with local information technology systems and an absence of technical support services. Usability was closely related to quality assurance as good design was felt to reduce training requirements and make devices safer to use. Improvements in this respect, especially when providing better functionality, facilitated adoption and diffusion.

For peer review only - http://bmjopen.bmj.com/site/about/guidelines.xhtml

Verbatim quotations to highlight the design and quality assurance barriers and facilitators to POCT adoption are provided in Text Box 6.

For peer review only

For peer review only - http://bmjopen.bmj.com/site/about/guidelines.xhtml

Verbatim quotations to highlight the usability and quality assurance barriers and facilitators to POCT adoption
"*doctors believe that they're endowed by God to be able to perform analytical measurements without any training or need for any quality assurance unfortunately this is simply not the case*" (R)

Text Box 6: **Verbatim quotations to highlight the usability and quality assurance barriers and facilitators to POCT adoption (R = Regulator)**

Patient Factors Facilitators

Many stakeholders emphasised the popularity of POCTs to patients. POCT strategies were often seen to improve the patient journey and provide patients with a better healthcare experience. This has led to patient demand for the implementation of POCTs in many clinical pathways, often encouraged by media coverage. In the management of chronic disease the ability of POCTs to give patients control of their own healthcare was a significant benefit of the technology. All these factors were seen as enablers to the use of POCTs and no patient associated barriers were identified. Verbatim quotations to highlight the patient related facilitators to POCT adoption are summarised in Text Box 7.

Verbatim quotations to highlight the patient factor facilitators to POCT adoption
"*patients themselves can see the advantage of it* [POCT]*"* (R)
"*it's quite difficult to say no to patients when they want a test*" (Cl)
"for me, it's been the most invigorating experience" (P)

Text Box 7: **Verbatim quotations to highlight the patient factor facilitators to POCT adoption (P = Patient, Cl = Clinician, R = Regulator)**

For peer review only - http://bmjopen.bmj.com/site/about/guidelines.xhtml

Other (Non–Financial) Resource Use Facilitators

This final theme covers the reduction in patient-healthcare interactions that POCTs can lead to, namely a reduction in outpatient appointments, hospital admissions and length of stay. No barriers were identified relating to other (non-financial) resource use. Verbatim quotations to highlight the other (non–financial) resource use facilitators to POCT adoption are provided in Text Box 8.

**Verbatim quotations to highlight the other (non-financial) resource use facilitators to POCT adoption**
"a lot of appointments are taken up with people coming back for results, a lot of resources, text messages; if you've got the results there and then and they've got the results they don't need to phone back and people are satisfied, they go away with a printout" (CI)
"a bigger, more strategic view, and that is this whole issue of (…) trying to keep patients out of hospital" (Com)
"*I am a patient in lots of things and I'd rather go and see my GP and then be able to say, "Well actually it's still a bit high, let's change it now," and that be done in the one visit than me going, me get a form, me have to go somewhere else to get my blood tests done*" (I)

Text Box 8: **Verbatim quotations to highlight the other (non-financial) resource use facilitators to POCT adoption (= Industry, CI = Clinician, Com = Commissioner)**

DISCUSSION

This study has identified 32 barriers and 27 facilitators to the adoption of POCT technology. It is unlikely that all of these will apply to one particular device but will be

For peer review only - http://bmjopen.bmj.com/site/about/guidelines.xhtml

dependent on factors such as the population, clinical pathway and experience of user. Many of the barriers and facilitators identified are closely related, for example the cost of the test against potential pathway cost savings. This implies that some of the barriers may be perceived rather than absolute and therefore often can be readily overcome with the right evidence, education or design modification. However, any barrier that is discovered during a device's development and seen as absolute, such as a lack of a real clinical need, should raise question as to whether there is value in continuing with development or whether a change of focus or design may be required. POCT barriers may also evolve over time and a degree of "future-proofing" may be required. This is particularly true in respect to POCT for infectious diseases that may be novel, mutate over time or develop resistance to antimicrobials. This is discussed further in a publication by European Joint Programming Initiative on Antimicrobial Resistance Transnational Working Group 'Antimicrobial Resistance - Rapid Diagnostic Tests' (JPIAMR AMR-RDT) who propose their 'mix-and-match' package for the implementation of POCT [16].

Each stakeholder group has different motivations for POCT adoption. Patient care is central to all; but clinicians may be influenced by their speciality, knowledge and the particular patients under their care, patients themselves respond to personal experiences, commissioners will likely have a defined focus on pathway benefits, as will regulators at a wider societal level and finally industry will be more focused on commercial aspects. In some circumstances patients have been shown to convey a more positive outlook regarding the adoption of POCT into healthcare systems compared to healthcare providers and industry [17].

Such different motivations may explain the variation in barriers and facilitators identified by each group. Whilst some were highlighted by most stakeholder groups (eg cost of test, siloed budgets, patient experience or training requirements) many others were more frequently picked up by one particular group such as industry's frustration at the lack of clinician buy-in or commissioner's focus on reducing

For peer review only - http://bmjopen.bmj.com/site/about/guidelines.xhtml

attendance at healthcare services, reconfiguration of pathways and facilitating local enhanced services. Reimbursement and funding mechanisms for new POCT will be test and setting specific and examples can be seen from the ways POCT testing for C-reactive protein can be established in primary care [5] and of financial drivers influencing practice [18]. When looking at stakeholder motivations it may be more useful to describe the barriers and facilitators as incentives or disincentives to adoption as often the implementation of a POCT strategy will bring advantages to some stakeholders but disadvantage (usually financially) others. Furthermore, incentives and attitudes may be influenced further by culture and international differences have been demonstrated in the preferred attributes of POCT [19].

This study supports and builds on the findings of the previous literature described in the introduction with the advantage of incorporating the diverse range of stakeholders included in the present study to allow for a comprehensive presentation of adoption influencers to be reported. The two most commonly cited barriers to POCT adoption in the current study were the cost of the test and associated training burden. This supports the results of the systematic review of Quinn et al [9] who reported the mostly commonly reported factors were related to economic issues, quality assurance and regulation. Quality assurance is a recurring concern in regards POCT where the end user may have limited or no training in the device itself or interpretation of the result.

When the emergent themes from this study are compared to the barriers identified in the interim report of the AAR [8] there is also good correlation. The themes from the AAR were *culture, data and evidence, finance and budgeting, systematic complexity and informational complaints, capacity/capability, dominance of the pharma paradigm and immaturity/uncertainty of landscape,* all of which were highlighted by the stakeholders we recruited in the present study. Stakeholders frequently used the evaluation of pharmaceuticals as a comparator, usually envying their established methodology and large budgets for evidence generation and marketing. This was echoed in the AAR interim report, where pharmaceutical industry was described as

For peer review only - http://bmjopen.bmj.com/site/about/guidelines.xhtml

dominating medical technology adoption given that NICE processes are based on pharmaceutical technology, horizon scanning is limited outside of pharmaceuticals and the challenging approval pathway for companion diagnostics.

This interview study highlighted several good examples of the use of POCTs but these tended to be small-scale pilot studies involving a small local practice or population. Furthermore, these were frequently short-term projects that were discontinued once funding came to an end, regardless of level of success. Such pilot schemes were rarely published or disseminated so other regions often have to re-invent POCT pathways leading to a repetitive evaluation processes and adding to the cost of establishing POCT services. This approach can be described as 'bottom-up' adoption and, with the one exception of faecal calprotectin, no examples were provided of 'top-down' adoption approach with central bodies driving adoption at a national level. Such'top-down' adoption may be able to significantly increase the number of appropriate POCTs that are adopted following the rigorous generation of high-quality evidence of patient and service benefits. National bodies including the National Institute for Health Research (NIHR) have recognised this approach and funded research groups to improve the evaluation of diagnostics to accelerate adoption. The NIHR Medtech In-vitro diagnostics Co-operatives [20] have multidisciplinary expertise and collaborate with industry and other stakeholders to analyse the impact of diagnostic to healthcare systems and generate data and evidence on a wide range of factors that contribute to the adoption of devices into clinical practice. The National Institute for Health and Care Excellence (NICE) has a health technology adoption team that aims to identify implementation barriers and promote technology adoption and includes diagnostic tests in its portfolio [21]. There are also examples of this approach being utilised by international consortiums [22].

This study has limitations. Given the resources available for this study a minimum of eight participants was included in each stakeholder group. Whilst the interviews were in depth and saturation appeared to have been reached increasing the number of

For peer review only - http://bmjopen.bmj.com/site/about/guidelines.xhtml

participants may have improved the breadth of findings from the study. Recruitment strategy varied between stakeholder groups and aimed to provide a representative sample of participants but due to the time burden associated with conducting semi-structured interviews a degree of convenience sampling was unavoidable and the study may be prone to recruitment bias. Furthermore, participants were often providing a perspective from their experiences within the UK National Health Service and it is unclear how transferable some of the barriers and facilitators identified would relate to different healthcare systems. One further limitation relating to this study is that the interviews were designed to develop the POCKET checklist with the identification of barriers and facilitators being undertaken as a secondary objective. This led to all POCTs being grouped together and many of the barriers and facilitators may be unique to a particular POCT testing strategy (such as self testing, primary care testing etc). However, relevant questions were included in the topic guide with a diverse group of stakeholders to comprehensively identify all the barriers and facilitators they have encountered with their expertise and experience.

This study provides an overview of the barriers and facilitators to the adoption of POCTs. In the most part these can be seen as incentives and disincentives. Widespread adoption of POCT technology can only occur if the disincentives can be predicted and mitigated. Therefore, these factors should be addressed as early as possible in the device development pathway to maximise the likelihood of widespread adoption. Qualitative research methodology including clinical needs assessments and stakeholder analyses can be used to assist this process and should all be undertaken early are regularly reviewed throughout the device development pathway.

Contibutorship Statement: GBH, JRH and JB conceived the project. GBH, JRH, JB and MN planned the project. JRH undertook the data collection. JRH and MN analysed the data. GBH, JRH, JB, and MN were involved in interpreting the data. All authors were involved in drafting the final manuscript.

For peer review only - http://bmjopen.bmj.com/site/about/guidelines.xhtml

Competing Interests: The authors declare no competing interest.

Data sharing: No data are available.

Funding: This research was supported by the National Institute for Health Research (NIHR) Diagnostic Evidence Co-operative London at Imperial College Healthcare NHS Trust. The views expressed are those of the authors and not necessarily those of the NHS, the NIHR or the Department of Health.

REFERENCES

1 Huddy JR, Ni MZ, Markar SR, *et al.* Point-of-care testing in the diagnosis of gastrointestinal cancers: Current technology and future directions. *World J Gastroenterol* 2015;**21**:4111. doi:10.3748/wjg.v21.i14.4111

2 Price CP. Point of care testing. *BMJ* 2001;**322**:1285–8. doi:10.1136/bmj.322.7297.1285

3 Rogers EM. *Diffusions of Innovations*. 1995.

4 Morris ZS, wooding S, Grant J. The answer is 17 years, what is the question: Understanding time lags in translational research. *J R Soc Med* Published Online First: 2011. doi:10.1258/jrsm.2011.110180

5 Huddy JR, Ni MZ, Barlow J, *et al.* Point-of-care C reactive protein for the diagnosis of lower respiratory tract infection in NHS primary care: A qualitative study of barriers and facilitators to adoption. *BMJ Open* Published Online First: 2016. doi:10.1136/bmjopen-2015-009959

6 Van Adrichem EJ, De Zande SCV, Dekker R, *et al.* Perceived barriers to and facilitators of physical activity in recipients of solid organ transplantation, a qualitative study. *PLoS One* Published Online First: 2016.

For peer review only - http://bmjopen.bmj.com/site/about/guidelines.xhtml

doi:10.1371/journal.pone.0162725

7 Witzel TC, Rodger AJ, Burns FM, *et al.* HIV self-testing among men who have sex with men (MSM) in the UK: A qualitative study of barriers and facilitators, intervention preferences and perceived impacts. *PLoS One* Published Online First: 2016. doi:10.1371/journal.pone.0162713

8 https://assets.publishing.service.gov.uk/government/uploads/system/uploads/attachment_data/file/471562/AAR_Interim_Report_acc.pdf (Accessed 3rd January 2021).

9 Quinn AD, Dixon D, Meenan BJ. Barriers to hospital-based clinical adoption of point-of-care testing (POCT): A systematic narrative review. *Crit Rev Clin Lab Sci* 2016;**53**:1–12. doi:10.3109/10408363.2015.1054984

10 Jones CH, Howick J, Roberts NW, *et al.* Primary care clinicians' attitudes towards point-of-care blood testing: a systematic review of qualitative studies. *BMC Fam Pract* 2013;**14**:117. doi:10.1186/1471-2296-14-117

11 Huddy JR, Ni M, Misra S, *et al.* Development of the Point-of-Care Key Evidence Tool (POCKET): A checklist for multi-dimensional evidence generation in point-of-care tests. *Clin Chem Lab Med* Published Online First: 2019. doi:10.1515/cclm-2018-1089

12 Tong A, Sainsbury P, Craig J. Consolidated criteria for reporting qualitative research (COREQ): A 32-item checklist for interviews and focus groups. *Int J Qual Heal Care* Published Online First: 2007. doi:10.1093/intqhc/mzm042

13 Huddy JR, Ni M, Mavroveli S, *et al.* A research protocol for developing a Point-Of-Care Key Evidence Tool 'POCKET': a checklist for multidimensional evidence reporting on point-of-care in vitro diagnostics: Figure 1. *BMJ Open* 2015;**5**:e007840. doi:10.1136/bmjopen-2015-007840

14 Brendish NJ, Poole S, Naidu VV, Mansbridge CT, Norton NJ, Wheeler H, Presland L, Kidd S, Cortes NJ, Borca F, Phan H, Babbage G, Visseaux B,

For peer review only - http://bmjopen.bmj.com/site/about/guidelines.xhtml

Ewings S, Clark TW. Clinical impact of molecular point-of-care testing for suspected COVID-19 in hospital (COV-19POC): a prospective, interventional, non-randomised, controlled study. Lancet Respir Med. 2020 Oct 8:S2213-2600(20)30454-9. doi: 10.1016/S2213-2600(20)30454-9. Epub ahead of print. PMID: 33038974; PMCID: PMC7544498

15 https://www.gov.uk/guidance/medical-devices-eu-regulations-for-mdr-and-ivdr (Accessed 3rd January 2021).

16 Hays JP, Mitsakakis K, Luz S, van Belkum A, Becker K, van den Bruel A, Harbarth S, Rex JH, Simonsen GS, Werner G, Di Gregori V, Lüdke G, van Staa T, Moran-Gilad J, Bachmann TT; JPIAMR AMR-RDT consortium. The successful uptake and sustainability of rapid infectious disease and antimicrobial resistance point-of-care testing requires a complex 'mix-and-match' implementation package. Eur J Clin Microbiol Infect Dis. 2019 Jun;38(6):1015-1022. doi: 10.1007/s10096-019-03492-4. Epub 2019 Feb 2. PMID: 30710202; PMCID: PMC6520316.

17 Kaman WE, Andrinopoulou ER, Hays JP. Perceptions of point-of-care infectious disease testing among European medical personnel, point-of-care test kit manufacturers, and the general public. Patient Prefer Adherence. 2013 Jun 19;7:559-77. doi: 10.2147/PPA.S44889. PMID: 23814465; PMCID: PMC3693915

18 https://bjgp.org/content/67/656/110.1 (Accessed 3rd January 2021).

19 Howick J, Cals JW, Jones C, Price CP, Plüddemann A, Heneghan C, Berger MY, Buntinx F, Hickner J, Pace W, Badrick T, Van den Bruel A, Laurence C, van Weert HC, van Severen E, Parrella A, Thompson M. Current and future use of point-of-care tests in primary care: an international survey in Australia, Belgium, The Netherlands, the UK and the USA. BMJ Open. 2014 Aug 8;4(8):e005611. doi: 10.1136/bmjopen-2014-005611. PMID: 25107438; PMCID: PMC4127935.

For peer review only - http://bmjopen.bmj.com/site/about/guidelines.xhtml

20 https://www.nihr.ac.uk/partners-and-industry/industry/access-to-expertise/medtech.htm (Accessed 3rd January 2021).

21 https://www.nice.org.uk/about/what-we-do/into-practice/adoption-team (Accessed 3rd January 2021).

22 https://www.karolinska.se/en/karolinska-university-hospital/Innovation/pippi/ (Accessed 3rd January 2021).

For peer review only

For peer review only - http://bmjopen.bmj.com/site/about/guidelines.xhtml

1 TABLES

Theme	Identified Barrier	Comm.	Clin.	Reg.	Industry	Patients	Total
**Clinical**	Inappropriate use of test	1	1	1	0	1	**4**
	Lack of clinical utility	1	4	0	2	0	**7**
	Reduced accuracy	0	2	2	0	0	**4**
	Discourages use of clinical judgement	0	1	0	1	0	**2**
**Cultural**	Lack of awareness	0	0	0	2	0	**2**
	Lack of clinician buy-in	0	0	0	6	1	**7**
	Resistance to change	4	0	1	2	0	**7**
	Need for service reconfiguration	5	0	0	2	0	**7**
	Lack of Accountability	0	0	0	0	1	**1**
**Evidence**	Cost of evidence generation	0	1	0	2	0	**3**
	Lack of evidence	2	0	1	0	0	**3**
	Lack of evaluation methodology	0	1	0	1	0	**2**
	Repetition of small scale local studies	0	0	0	3	0	**3**
	Rate of technology development	0	0	1	0	0	**1**

For peer review only - http://bmjopen.bmj.com/site/about/guidelines.xhtml

**Financial**	Commissioning	1	0	0	0	3	**4**
	Cost of test	5	3	1	3	2	**14**
	Lack of investment in new technology	0	1	0	3	0	**4**
	Payer identification	1	0	0	2	0	**3**
	Reimbursement	0	0	2	0	0	**2**
	Siloed budgets	1	1	1	1	0	**4**
**Organisational**	Asset control	0	0	1	0	0	**1**
	Duration of consultation	1	2	1	0	0	**4**
	Increased workload	2	1	0	1	0	**3**
	Threat to laboratory services	4	0	1	3	0	**8**
	Reduced throughput	0	0	1	0	0	**1**
	Regulation	0	0	0	2	0	**2**
	Trust Autonomy	0	0	0	2	0	**2**
**Design and Quality Assurance**	Maintenance Requirements	1	0	1	0	0	**2**
	Training Burden	2	6	4	2	0	**14**
	Poor Usability	0	0	1	1	0	**2**

For peer review only - http://bmjopen.bmj.com/site/about/guidelines.xhtml

	Lack of connectivity	1	0	0	1	0	**2**
	Lack of technical support	0	0	1	0	0	**1**

Table 1: **Identified barriers to the adoption of POCT and frequencies of stakeholders who highlighted each by stakeholder group (Comm – commissioners, Clin – clinicians, Reg - Regulators)**

For peer review only - http://bmjopen.bmj.com/site/about/guidelines.xhtml

Theme	Identified Facilitator	Comm.	Clin.	Reg.	Industry	Patients	Total
Clinical	Improving doctor-patient communication	2	0	0	0	0	**2**
	Improving patient care	0	2	1	2	0	**5**
	Time to result	0	4	1	2	0	**7**
	More effective triage	0	0	0	1	0	**1**
	Reduced time to treatment	4	0	5	1	0	**10**
Cultural	Word of mouth	0	0	0	0	4	**4**
	Incentives to adopt	0	1	0	1	0	**2**
	Key opinion leaders	1	0	0	0	2	**3**
	NHS and industry partnerships	1	1	0	1	0	**3**
Evidence	Availability of patient level data	1	0	1	0	0	**2**
	Successful pilot data (local level)	0	0	0	2	0	**2**
Financial	Cost savings	1	0	1	1	0	**3**
	Improved efficiency	3	2	1	2	0	**8**
Organisational	Connectivity	0	0	0	0	2	**2**
	Flow	0	0	0	0	2	**2**

For peer review only - http://bmjopen.bmj.com/site/about/guidelines.xhtml

	Government policy	0	0	0	1	0	**1**
	POCT use in local enhanced services	3	0	0	0	0	**3**
	Locality of diagnostics	0	2	0	2	0	**4**
	Support from central pathology laboratories	2	1	1	1	0	**5**
**Design and Quality Assurance**	Good design and functionality	0	1	0	1	0	**2**
	Usability	1	0	1	0	0	**2**
**Patient Factors**	Patient demand	0	1	1	0	0	**2**
	Patient experience/patient journey	3	1	2	2	0	**8**
	Patient ownership	3	0	1	1	7	**12**
**Resource Use**	Reduced attendance to healthcare services	6	1	1	0	0	**8**
	Reduced hospital admissions	0	0	1	1	0	**2**
	Reduced length of stay	0	0	1	1	0	**2**

Table 2 Identified facilitators to the adoption of POCT and frequencies of stakeholders who highlighted each by stakeholder group (Comm – commissioners, Clin – clinicians, Reg - Regulators)

For peer review only - http://bmjopen.bmj.com/site/about/guidelines.xhtml

For peer review only

For peer review only - http://bmjopen.bmj.com/site/about/guidelines.xhtml

APPENDIX

Appendix 1: POCKET Interview prompt (initial version)

Section 1: Point-of-Care Tests

Clinical Needs Assessment

Example Introductory Statement
"Point-of–care diagnostic tests allow for diagnostic tests to be performed at the bedside, in the clinic or in the patient's home. With advancing diagnostic technologies new point of care tests can give rapid or instant results allowing for real-time clinical decision making in many diseases."

1. What is your opinion of point-of-care diagnostic tests?

   Prompt if required for advantages/disadvantages

2. Where are they required? Why?

3. How can we know where POC diagnostic tests would give most benefit?

   a. (clinical needs assessment/unmet clinical need) Explore unmet needs.

4. Please mark which of the following are useful to determine unmet clinical needs:
   a. Systematic literature reviews
   b. Expert opinion
   c. Patient opinion
   d. Competition analysis
   e. Other

5. Who should be establishing unmet needs?

Section 2: Evidence and Evaluation

1. Once a new point of care diagnostic test has been produced what should the next steps be?

   Prompts: How should we evaluate these devices? Why?

2. What are the goals of evidence generation in new point-of-care diagnostic tests?

1. What evidence best leads to adoption?

2. Do you have any examples of devices that have been adopted easily or with particular difficulty. What caused this? Prompt: home coagulation checking.

3. Please rank the evidence you regard as vital, useful, not required or unsure for the implementation of point of care devices that will be used **in healthcare institutions**?

For peer review only

a. Clinical trials
b. Usability assessment
c. Design Evaluation
d. Patient experience
e. Clinical needs assessment
f. Clinical Pathway Mapping
g. Patient safety/Risk Assessment
h. Economic Analysis
i. Stakeholder Analysis
j. Barriers to adoption
k. Decision Analysis
l. Other

4. Please rank the evidence you regard as vital, useful, not required or unsure for the implementation of point of care devices that will be used **in patient's homes**?

    a. Clinical trials
    b. Usability assessment
    c. Design Evaluation
    d. Patient experience
    e. Clinical needs assessment
    f. Clinical Pathway Mapping
    g. Patient safety/Risk Assessment
    h. Economic Analysis
    i. Stakeholder Analysis
    j. Barriers to adoption
    k. Decision Analysis
    l. Other

5. Where should this evidence come from?
    a. Industry
    b. Academia
    c. Industry/academia partnerships
    d. Consultancy firms
    e. Other

6. What incentives can enhance the adoption of point-of-care test devices?

For peer review only

Validity

Example Introductory Statement
"We will now discuss clinical validity or the accuracy of a diagnostic test."

1. What should evidence on clinical validity demonstrate?

2. Please rank the usefulness of these sources of evidence for clinical validity?

    a. Randomised controlled trial evidence
    b. Diagnostic Accuracy Studies (Case-control)
    c. Linked Evidence Approaches
    d. Bayesian Networks
    e. Other approach

3. What if there is no reference standard?

"Point-of-care tests are often seen as a disruptive innovation, by which I mean that the introduction of the test disturbs the established clinical pathway. This can be demonstrated by clinical pathway modeling to predict the impact of implementation."

4. Should new tests be modeled against current practice or best practice?

5. How translatable is this evidence between different institutions or different healthcare systems?

6. Is a trade off in accuracy acceptable for the convenience of point-of-care tests?

7. Any other thoughts regarding the accuracy or validity of POC tests?

<u>Usability</u>

Introductory Statement
"Point-of-care tests are often performed away from central laboratories or in the patient's home and therefore trained laboratory personnel are not present and those using the test will have limited training. Therefore, these devices need to be user-friendly."

1. What are your thoughts on the usability of point-of care diagnostic test?

2. Should this be tested? How?

3. Is evidence demonstrating the usability of a device important for implementation?

4. How should this be presented?

5. What usability assessments are you aware of?

6. Is evidence on usability required that is over and above what is required for regulatory approval (FDA/CE marking)?

7. How does patient safety impact on implementation of new tests?

    a. Prompt if required re risk assessment

8. Any other thoughts on the usability assessment of point-of-care diagnostic tests?

<u>Economic Analysis</u>

Introductory Statement
"Healthcare budgets are a finite resource and cost-effectiveness needs to be demonstrated to justify the use of new diagnostic tests."

For peer review only

1. What do you think gives value to new point-of-care diagnostic tests?

2. How is this measured?

3. What economic information would benefit the implementation of point-of-care diagnostic tests?

4. Are modeling techniques reliable? Why?

7. Are quality adjusted life years (QALY) a useful and sufficient measurement of value in the evaluation of diagnostic tests?

8. Any other thoughts regarding the economic analysis of point-of-care diagnostic tests?

Patient Experience

Introductory statement
"Patients are the reason why diagnostic tests are performed so when engaging with patients regarding new technologies…"

1. How should patient experience and opinion be incorporated into the evaluation of new point-of-care diagnostic tests?

2. What evidence should patients have in new diagnostic tests?

Section 3: Adoption

Barriers

Example Introductory statement
"Even with good evidence some point-of care-technologies fail or are slow to be implemented."

1. What are the challenges in generating evidence to justify the implementation of new point-of-care diagnostic tests?

1. How can these be overcome?

2. Is there any other evidence you feel would assist in the implementation of new point-of-care diagnostic tests?

3. Are there any other barriers to the implementation of new point-of-care diagnostic tests?

4. What can the NIHR Diagnostic Evidence Co-operatives do to help you make decisions?

5. NICE/Industry/CCG: As a partner to the Diagnostic Evidence Co-operatives, how do you envisage us working together?

Efficient Evidence Generation

For peer review only

Example Introductory Statement
"Effective study design may allow evidence in differing domains to be gathered more efficiently."

1. How can we be more efficient in the evaluation process of point-of-care diagnostic tests?

2. What changes would you like to see?

3. Are you aware of Bayesian Networks being used to combine sources of evidence?

4. Any other thoughts or comments regarding point-of-care tests and evidence generation?

For peer review only

COREQ checklist for semi-structured interview checklist [14]

Domain 1: Research team and reflexivity			
Personal Characteristics			
1	Interviewer/facilitator	Which author/s conducted the interview or focus group?	JRH (Page 6, line 27)
2	Credentials	What were the researcher's credentials? E.g. PhD, MD	BMedSci BMBS MRCS (Not included in manuscript)
3	Occupation	What was their occupation at the time of the study?	Clinical Research Fellow (Not included in manuscript)
4	Gender	Was the researcher male or female?	Male (Not included in manuscript)
5	Experience and training	What experience or training did the researcher have?	This was the first qualitative study undertaken by the researcher. A co-researcher with experience in qualitative research supported the work and the primary researcher undertook a course in qualitative research methodology during the study. (Not included in manuscript)
Relationship with participants			
6	Relationship established	Was a relationship established prior to study commencement?	There was no prior relationship with 38/43 participants. The researcher through previous academic collaboration knew five participants. (Not included in manuscript)
7	Participant knowledge of the interviewer	What did the participants know about the researcher? e.g. personal goals, reasons for doing the research	Participants knew that the work was being undertaken by a clinical research fellow towards a PhD under the remit of the NIHR Diagnostic Evidence Co-operative. Participants knew the overall aims of the project. (Not included in manuscript)
8	Interviewer characteristics	What characteristics were reported about the interviewer/facilitator? e.g. Bias, assumptions, reasons and interests in the research topic	As above (Not included in manuscript)
Domain 2: study design			

For peer review only

Theoretical framework			
9	Methodological orientation and Theory	What methodological orientation was stated to underpin the study? e.g. grounded theory, discourse analysis, ethnography, phenomenology, content analysis	This study was based on grounded theory (page 6, line 15).
Participant selection			
10	Sampling	How were participants selected? e.g. purposive, convenience, consecutive, snowball	Sampling was dependent on stakeholder group. Purposive sampling was intended but convenience sampling undertaken when recruitment did not allow (described in protocol manuscript referenced in text).
11	Method of approach	How were participants approached? e.g. face-to-face, telephone, mail, email	Email (described in protocol manuscript referenced in text).
12	Sample size	How many participants were in the study?	43 participants were included in total with 8-10 in each stakeholder group (page 7, lines 19-21) .
13	Non-participation	How many people refused to participate or dropped out? Reasons?	This is not known for the patients, industry and regulators group as participants were approached by a body external to the research group. Two invited clinicians did not participate (one did not reply to invitation and one did not have mutual availability for interview). Twenty commissioners declined to participate (two declined as did not have experience of subject and 18 did not reply to invitation).
Setting			
14	Setting of data collection	Where was the data collected? e.g. home, clinic, workplace	Workplace. The exception to this was patients were data was collected by telephone interview with the patient at home (not detailed in manuscript).
15	Presence of non-participants	Was anyone else present besides the participants and researchers?	No, with the exception of the two interviews that were undertaken with two participants (not detailed in manuscript).
16	Description of sample	What are the important characteristics of the sample? e.g. demographic data, date	This data is not available.
Data collection			

For peer review only

17	Interview guide	Were questions, prompts, guides provided by the authors? Was it pilot tested?	Yes (see appendix).
18	Repeat interviews	Were repeat interviews carried out? If yes, how many?	No (described in protocol manuscript referenced in text).
19	Audio/visual recording	Did the research use audio or visual recording to collect the data?	Audio recording (described in protocol manuscript referenced in text).
20	Field notes	Were field notes made during and/or after the interview or focus group?	No (described in protocol manuscript referenced in text).
21	Duration	What was the duration of the interviews or focus group?	Median length of 31 minutes and 21 seconds (range 11 minutes and 50 seconds - 58 minutes and 20 seconds) (page 7, lines 25-26).
22	Data saturation	Was data saturation discussed?	Yes (page 7, lines 10-14).
23	Transcripts returned	Were transcripts returned to participants for comment and/or correction?	No, the methodology allowed for clarification of comments but this was not necessary during analysis (not detailed in manuscript).
Domain 3: analysis and findings			
Data analysis			
24	Number of data coders	How many data coders coded the data?	Two (page 7, line 7).
25	Description of the coding tree	Did authors provide a description of the coding tree?	Yes (page 7, lines 27- page 8, line 2).
26	Derivation of themes	Were themes identified in advance or derived from the data?	Some themes were identified in advance but these were modified during the analysis process (described in protocol manuscript. This is referenced in the manuscript).
27	Software	What software, if applicable, was used to manage the data?	NVivo (page 7, line 12).
28	Participant checking	Did participants provide feedback on the findings?	No (page 7, lines 6-7)
Reporting			
29	Quotations presented	Were participant quotations presented to illustrate the themes / findings?<br><br>Was each quotation identified? e.g. participant number	Yes (page 7, line 16 – page 18, line 12)<br><br>Yes – stakeholder group identified (page 7, line 16 – page 18, line 12)

For peer review only

30	Data and findings consistent	Was there consistency between the data presented and the findings?	Yes (page 7, line 16 – page 18, line 12)
31	Clarity of major themes	Were major themes clearly presented in the findings?	Yes (page 7, line 16 – page 18, line 12)
32	Clarity of minor themes	Is there a description of diverse cases or discussion of minor themes?	Yes (page 7, line 16 – page 18, line 12)

For peer review only
